# Association of Intima‐Media Thickness Measured at the Common Carotid Artery With Incident Carotid Plaque: Individual Participant Data Meta‐Analysis of 20 Prospective Studies

**DOI:** 10.1161/JAHA.122.027657

**Published:** 2023-06-10

**Authors:** Lena Tschiderer, Lisa Seekircher, Raffaele Izzo, Costantino Mancusi, Maria V. Manzi, Damiano Baldassarre, Mauro Amato, Elena Tremoli, Fabrizio Veglia, Tomi‐Pekka Tuomainen, Jussi Kauhanen, Ari Voutilainen, Bernhard Iglseder, Lars Lind, Tatjana Rundek, Moise Desvarieux, Akihiko Kato, Eric de Groot, Gülay Aşçi, Ercan Ok, Stefan Agewall, Joline W. J. Beulens, Christopher D. Byrne, Philip C. Calder, Hertzel C. Gerstein, Paolo Gresele, Gerhard Klingenschmid, Michiaki Nagai, Michael H. Olsen, Grace Parraga, Maya S. Safarova, Naveed Sattar, Michael Skilton, Coen D. A. Stehouwer, Heiko Uthoff, Michiel A. van Agtmael, Amber A. van der Heijden, Dorota A. Zozulińska‐Ziółkiewicz, Hyun‐Woong Park, Moo‐Sik Lee, Jang‐Ho Bae, Oscar Beloqui, Manuel F. Landecho, Matthieu Plichart, Pierre Ducimetiere, Jean Philippe Empana, Lena Bokemark, Göran Bergström, Caroline Schmidt, Samuela Castelnuovo, Laura Calabresi, Giuseppe D. Norata, Liliana Grigore, Alberico Catapano, Dong Zhao, Miao Wang, Jing Liu, M. Arfan Ikram, Maryam Kavousi, Michiel L. Bots, Michael J. Sweeting, Matthias W. Lorenz, Peter Willeit, Proof‐ATHERO Study Group

**Affiliations:** ^1^ Institute of Health Economics Medical University of Innsbruck Innsbruck Austria; ^2^ Department of Advanced Biomedical Sciences Federico II University Naples Italy; ^3^ Department of Medical Biotechnology and Translational Medicine University of Milan Milan Italy; ^4^ Centro Cardiologico Monzino Stituto di Ricovero e Cura a Carattere Scientifico Milan Italy; ^5^ Maria Cecilia Hospital Cotignola (Ravenna) Italy; ^6^ Klinik für Neurologie Krankenhaus Nordwest Frankfurt am Main Germany; ^7^ Department of Clinical Sciences, Danderyd Hospital Division of Cardiology Karolinska Institutet Stockholm Sweden; ^8^ Institute of Public Health and Clinical Nutrition University of Eastern Finland Kuopio Finland; ^9^ Department of Geriatric Medicine Gemeinnützige Salzburger Landeskliniken Betriebsgesellschaft GmbH Christian‐Doppler‐Klinik Salzburg Austria; ^10^ Department of Geriatric Medicine Paracelsus Medical University Salzburg Austria; ^11^ Department of Medicine Uppsala University Uppsala Sweden; ^12^ Department of Neurology University of Miami Miller School of Medicine Miami FL; ^13^ Department of Epidemiology, Mailman School of Public Health Columbia University New York NY; ^14^ METHODS Core, Centre de Recherche Epidémiologie et Statistique Paris Sorbonne Cité Institut National de la Santé et de la Recherche Médicale Unité Mixte de Recherche 1153 Paris France; ^15^ Blood Purification Unit Hamamatsu University Hospital Hamamatsu Japan; ^16^ Imagelabonline and Cardiovascular Erichem the Netherlands; ^17^ Department of Gastroenterology and Hepatology, Amsterdam University Medical Center–Academic Medical Centre Amsterdam the Netherlands; ^18^ Nephrology Department Ege University School of Medicine Bornova‐Izmir Turkey; ^19^ Institute of Clinical Sciences University of Oslo Oslo Norway; ^20^ Department of Epidemiology and Data Science, Amsterdam University Medical Center–Location Vrije Universiteit Medical Center Amsterdam the Netherlands; ^21^ School of Human Development and Health, Faculty of Medicine University of Southampton Southampton UK; ^22^ Southampton National Institute for Health and Care Research, Biomedical Research Centre University Hospital Southampton Southampton UK; ^23^ Department of Medicine and Population Health Research Institute McMaster University Hamilton Ontario Canada; ^24^ Hamilton General Hospital Hamilton Ontario Canada; ^25^ Division of Internal and Cardiovascular Medicine, Department of Medicine and Surgery University of Perugia Perugia Italy; ^26^ Department of Neurology Medical University of Innsbruck Innsbruck Austria; ^27^ Department of Internal Medicine General Medicine and Cardiology, Hiroshima City Asa Hospital Hiroshima Japan; ^28^ Department of Internal Medicine, Holbaek Hospital University of Southern Denmark Odense Denmark; ^29^ Department of Medical Biophysics, Robarts Research Institute Western University London ON Canada; ^30^ Department of Cardiovascular Medicine University of Kansas Medical Center Kansas City KS; ^31^ British Heart Foundation Glasgow Cardiovascular Research Centre University of Glasgow Glasgow UK; ^32^ Charles Perkins Centre, Faculty of Medicine and Health University of Sydney Sydney NSW Australia; ^33^ Department of Internal Medicine and Cardiovascular Research Institute Maastricht Maastricht University Medical Centre Maastricht the Netherlands; ^34^ Department of Angiology University Hospital Basel Basel Switzerland; ^35^ Department of Internal Medicine Amsterdam University Medical Center, Vrije Universiteit Amsterdam the Netherlands; ^36^ Department of General Practice, Amsterdam University Medical Center–Location Vrije Universiteit Medical Center Amsterdam the Netherlands; ^37^ Department of Internal Medicine and Diabetology Poznan University of Medical Sciences Poznan Poland; ^38^ Division of Cardiology, Department of Internal Medicine Chungnam National University Sejong Hospital Sejong‐si South Korea; ^39^ Department of Preventive Medicine, College of Medicine Konyang University Daejeon South Korea; ^40^ Department of Occupational and Environmental Medicine Konyang University Hospital Daejeon South Korea; ^41^ Heart Center, Konyang University Hospital Daejeon South Korea; ^42^ Department of Cardiology Konyang University College of Medicine Daejeon South Korea; ^43^ Department of Internal Medicine University Clinic of Navarra Navarra Spain; ^44^ Paris Cardiovascular Research Centre University Paris Descartes Paris France; ^45^ Fondation Santé Service, Hospital at Home Levallois‐Perret France; ^46^ Faculty of Medicine University Paris Descartes Paris France; ^47^ Wallenberg Laboratory for Cardiovascular Research University of Gothenburg Gothenburg Sweden; ^48^ Department of Molecular and Clinical Medicine, Institute of Medicine, Sahlgrenska Academy University of Gothenburg Gothenburg Sweden; ^49^ Department of Clinical Physiology Sahlgrenska University Hospital, Region Västragötaland Gothenburg Sweden; ^50^ Centro Dislipidemie, Aziende Socio Sanitarie Territoriali Grande Ospedale Metropolitano Niguarda Milan Italy; ^51^ Department of Pharmacological and Biomolecular Sciences University of Milan Milan Italy; ^52^ Società Italiana per lo Studio dell'Aterosclerosi Center for the Study of Atherosclerosis, Bassini Hospital Cinisello Balsamo Italy; ^53^ Stituto di Ricovero e Cura a Carattere Scientifico Multimedica Milan Italy; ^54^ Department of Epidemiology, Beijing Anzhen Hospital Capital Medical University Beijing China; ^55^ Department of Epidemiology Erasmus University Medical Center Rotterdam the Netherlands; ^56^ Julius Center for Health Sciences and Primary Care University Medical Center Utrecht Utrecht the Netherlands; ^57^ Department of Health Sciences University of Leicester Leicester UK; ^58^ Department of Public Health and Primary Care University of Cambridge Cambridge UK; ^59^ Department of Neurology Goethe University Frankfurt am Main Germany

**Keywords:** carotid intima‐media thickness, carotid plaque, individual participant data meta‐analysis, prospective studies, Epidemiology, Cardiovascular Disease, Ultrasound

## Abstract

**Background:**

The association between common carotid artery intima‐media thickness (CCA‐IMT) and incident carotid plaque has not been characterized fully. We therefore aimed to precisely quantify the relationship between CCA‐IMT and carotid plaque development.

**Methods and Results:**

We undertook an individual participant data meta‐analysis of 20 prospective studies from the Proof‐ATHERO (Prospective Studies of Atherosclerosis) consortium that recorded baseline CCA‐IMT and incident carotid plaque involving 21 494 individuals without a history of cardiovascular disease and without preexisting carotid plaque at baseline. Mean baseline age was 56 years (SD, 9 years), 55% were women, and mean baseline CCA‐IMT was 0.71 mm (SD, 0.17 mm). Over a median follow‐up of 5.9 years (5th–95th percentile, 1.9–19.0 years), 8278 individuals developed first‐ever carotid plaque. We combined study‐specific odds ratios (ORs) for incident carotid plaque using random‐effects meta‐analysis. Baseline CCA‐IMT was approximately log‐linearly associated with the odds of developing carotid plaque. The age‐, sex‐, and trial arm–adjusted OR for carotid plaque per SD higher baseline CCA‐IMT was 1.40 (95% CI, 1.31–1.50; *I* ^2^=63.9%). The corresponding OR that was further adjusted for ethnicity, smoking, diabetes, body mass index, systolic blood pressure, low‐ and high‐density lipoprotein cholesterol, and lipid‐lowering and antihypertensive medication was 1.34 (95% CI, 1.24–1.45; *I* ^2^=59.4%; 14 studies; 16 297 participants; 6381 incident plaques). We observed no significant effect modification across clinically relevant subgroups. Sensitivity analysis restricted to studies defining plaque as focal thickening yielded a comparable OR (1.38 [95% CI, 1.29–1.47]; *I* ^2^=57.1%; 14 studies; 17 352 participants; 6991 incident plaques).

**Conclusions:**

Our large‐scale individual participant data meta‐analysis demonstrated that CCA‐IMT is associated with the long‐term risk of developing first‐ever carotid plaque, independent of traditional cardiovascular risk factors.

**Nonstandard Abbreviations and Acronyms**
CCA: common carotid artery
cIMT: carotid intima‐media thickness
IMT: intima‐media thickness
Proof‐ATHERO: Prospective Studies of Atherosclerosis

**Clinical Perspective**

**What Is New?**

- This study, based on participant‐level data on 21 494 individuals from 20 studies, performed the most comprehensive analysis of the relationship between carotid intima‐media thickness and incident carotid plaque available to date.
- Carotid intima‐media thickness measured at the common carotid artery was positively and approximately log‐linearly associated with the long‐term risk of developing carotid plaque.
- This association was independent of cardiovascular risk factors and was robust across several subgroup and sensitivity analyses.

**What Are the Clinical Implications?**

- This study provides evidence for the role of carotid intima‐media thickness as a risk marker for atherosclerotic disease, which may help to identify individuals at risk of developing advanced atherosclerotic lesions earlier.

Carotid intima‐media thickness (cIMT) and carotid plaque are commonly used imaging markers for the development and progression of atherosclerosis, the pathophysiological mechanism underlying most cardiovascular diseases (CVDs). Both cIMT and carotid plaque can be measured noninvasively using high‐resolution B‐mode ultrasound. The 2 markers have been implicated in cardiovascular risk assessment, showing robust associations with common cardiovascular risk factors,^1^, ^2^, ^3^ atherosclerosis elsewhere in the arterial system,^4^ and the risk of developing a CVD event.^5^, ^6^, ^7^, ^8^

Observational studies investigating the association between cIMT and carotid plaque have produced variable results. Although cross‐sectional studies consistently showed that elevated cIMT values are associated with presence of carotid plaque,^9^, ^10^, ^11^, ^12^, ^13^, ^14^, ^15^, ^16^, ^17^ longitudinal studies investigating the association of baseline cIMT values with incident carotid plaque have yielded mixed results.^14^, ^16^, ^17^, ^18^, ^19^, ^20^, ^21^, ^22^, ^23^, ^24^, ^25^, ^26^, ^27^, ^28^, ^29^ We have recently summarized the evidence on this topic in a literature‐based meta‐analysis that involved data from 7 general population cohort studies with a total of 9341 participants without preexisting carotid plaque.^30^ In aggregate, it showed that individuals in the top quartile compared with those in the bottom quartile of baseline common carotid artery intima‐media thickness (CCA‐IMT) had a relative risk of 1.78 (95% CI, 1.53–2.07) of developing first‐ever carotid plaque. Because this meta‐analysis relied on literature‐based aggregated data, it was unable to apply consistent statistical methods with respect to adjustment for confounders, participant‐level inclusion criteria, and uniform definitions of exposure and outcome variables. In addition, it could only inspect effects of potential effect modifiers across averaged values or percentages, making it vulnerable to ecological fallacy.^31^

To address this gap in knowledge, we conducted an individual participant data meta‐analysis of 21 494 participants from 20 studies within the Proof‐ATHERO (Prospective Studies of Atherosclerosis) consortium with the aim of precisely characterizing the association of baseline CCA‐IMT with the risk of developing a first‐ever carotid plaque during follow‐up.

## METHODS

The data sets supporting the conclusions of this article are not made publicly available because of legal restrictions arising from the data distribution policy of the Proof‐ATHERO collaboration and from the bilateral agreements between the consortium’s coordinating center and participating studies, but they may be requested directly from individual study investigators. Studies that shared individual participant data have obtained informed consent of the study participants and ethical approval by their respective institutional review boards. This study conforms to the Preferred Reporting Items for Systematic Review and Meta‐Analyses of individual participant data (PRISMA‐IPD) guidelines.^32^ The PRISMA‐IPD checklist is provided in Table S1.

### Data Collection and Eligibility Criteria

Data were sought from the Proof‐ATHERO consortium; a detailed description of this collaboration has been published elsewhere.^33^ For inclusion in the current analysis, participants were required to have data pertaining to (1) baseline CCA‐IMT and (2) carotid plaque status (yes versus no) at baseline and at least at one visit during follow‐up. The baseline visit was defined as the first visit, at which carotid plaque status was available, and follow‐up as subsequent visits. We excluded participants with a baseline history of CVD (defined as coronary heart disease or stroke) or preexisting carotid plaque at baseline from the analysis. Furthermore, to avoid overfitting and convergence issues of statistical models, we excluded studies that recorded <20 events of incident carotid plaque. Moreover, we searched the literature for additional prospective studies on the association of baseline CCA‐IMT with incident carotid plaque in individuals free of carotid plaque at baseline that were published until December 1, 2022. We used the search terms (“intima‐media thickness” [all fields] OR “IMT” [all fields] OR “intima media thickness” [all fields] AND “plaque” [all fields] AND “incident” [all fields] OR “prospective” [all fields]) in PubMed and TS=(“intima‐media thickness” OR “IMT” OR “intima media thickness”) AND TS=(“plaque” AND [“incident” OR “prospective”]) in Web of Science.

### Ascertainment of CCA‐IMT and Carotid Plaque

Details on the study‐specific definitions of CCA‐IMT and carotid plaque are provided in Table S2 and have been described previously.^33^ In quantifying CCA‐IMT, we gave preference to mean CCA‐IMT values or, alternatively, used maximum CCA‐IMT. When studies provided cIMT measurements at several locations of the CCA (ie, near and far wall, left and right side, and different insonation angles), we used the arithmetic mean of all available values. When measuring cIMT, most studies focused on a 10‐mm long segment at the distal part of the CCA (Table S2 and Figure S1). Incident carotid plaque was defined as the development of first‐ever plaque during follow‐up in any segment of the carotid artery (ie, left or right CCA, carotid bifurcation, or internal carotid artery). Fourteen studies (70%) defined carotid plaque as focal thickening, and some others relied on different thresholds of cIMT (Table S2).

### Statistical Analysis

Statistical analyses were conducted according to a predefined analysis plan. We calculated odds ratios (ORs) for incident plaque using a 2‐stage approach. We first estimated ORs within each study separately, and then combined study‐specific ORs using random‐effects meta‐analysis using the method of moments procedure of DerSimonian and Laird. Between‐studies heterogeneity was quantified with the *I* ^2^ statistics.^34^ We conducted complete‐case analyses, if not stated otherwise.

In the primary analysis, we used logistic regression models to estimate ORs for incident plaque per SD higher level of CCA‐IMT, defining the SD of CCA‐IMT within each study separately. The CCA‐IMT distribution was checked for normality by visually inspecting quantile‐quantile plots. We report ORs (1) adjusted for age, sex, and trial arm; and (2) further adjusted progressively for ethnicity, smoking, history of diabetes, body mass index, systolic blood pressure, low‐density lipoprotein cholesterol, high‐density lipoprotein cholesterol, lipid‐lowering medication, antihypertensive medication, estimated glomerular filtration rate, and hs‐CRP (high‐sensitivity C‐reactive protein). We also conducted analyses that expressed ORs per 0.1‐mm higher level of baseline CCA‐IMT. To inspect the shape of association between baseline CCA‐IMT and incident plaque, we calculated ORs across study‐specific CCA‐IMT quintiles, pooled them using multivariate random‐effects meta‐analysis,^35^ plotted them against the mean CCA‐IMT value within each quintile, and added the best‐fitting line through the OR estimates. We evaluated log linearity of the association between baseline CCA‐IMT and incident carotid plaque by visually inspecting whether OR estimates lie on the corresponding best‐fitting lines. In this analysis, we used floating absolute risks^36^ to calculate 95% CIs for quintile groups (including the reference group), thereby enabling head‐to‐head comparisons between effect sizes of any 2 of the quintiles.

We also investigated effect modification with formal tests of interaction across clinically relevant predefined variables (ie, age, sex, lipid‐lowering medication, and low‐density lipoprotein cholesterol at baseline and development of CVD during follow‐up). We used meta‐regression^37^ to test for differences by selected study‐level characteristics (ie, study type and type of CCA‐IMT measurement). In subgroup analyses, we applied Bonferroni correction^38^ to account for multiple testing (ie, *P* values ≤0.0071 [0.05/7 tests] were deemed as statistically significant). In addition, we investigated whether ORs varied by median duration of follow‐up using meta‐regression.^37^ Moreover, we conducted sex‐specific analyses and estimated pooled ORs separately for women and men.

Finally, we conducted sensitivity analyses that: (1) took into account the time to plaque development by use of Cox regression (after ensuring that the proportional hazards assumption was met on the basis of Schoenfeld residuals and the graphical inspection of log[−log] plots), estimating the date of carotid plaque development as the visit at which carotid plaque had first been detected or, alternatively, as the midpoint between this and the preceding visit; (2) used long‐term average CCA‐IMT values (“usual levels”) estimated with regression calibration^39^ on the basis of repeated CCA‐IMT measurements over time; (3) used within‐study multiple imputation of missing values suggested by Burgess et al^40^ (ie, imputed sporadically missing values in each study separately [80 data sets] before applying the Rubin rule and then combining study‐specific effect sizes with random‐effects meta‐analysis); (4) omitted participants with a large CCA‐IMT value (>1.5 mm), which could be indicative of undetected carotid plaque; and (5) omitted studies that had defined carotid plaque as CCA‐IMT above a specific threshold rather than as focal thickening. We additionally conducted a separate sensitivity analysis that compared the association of baseline CCA‐IMT with carotid plaque development at the same side of the neck (ie, right CCA‐IMT with right carotid plaque and left CCA‐IMT with left carotid plaque) and at the opposite side of the neck (ie, right CCA‐IMT with left carotid plaque and left CCA‐IMT with right carotid plaque).

In addition, we meta‐analyzed the results of the studies from the Proof‐ATHERO consortium with the studies we found in the literature for which we were not able to obtain individual participant data. We focused on the Proof‐ATHERO studies included in our multivariable‐adjusted meta‐analysis to enhance the comparability to the studies from the literature. Again, we meta‐analyzed ORs for incident carotid plaque per SD higher baseline CCA‐IMT using random‐effects meta‐analysis.

All statistical tests were 2‐sided, and we deemed *P*≤0.05 as statistically significant, unless specified otherwise. Statistical analyses were conducted using Stata version 15.1 (StataCorp).

## RESULTS

### Contributing Data and Study Characteristics

**Figure 1:**
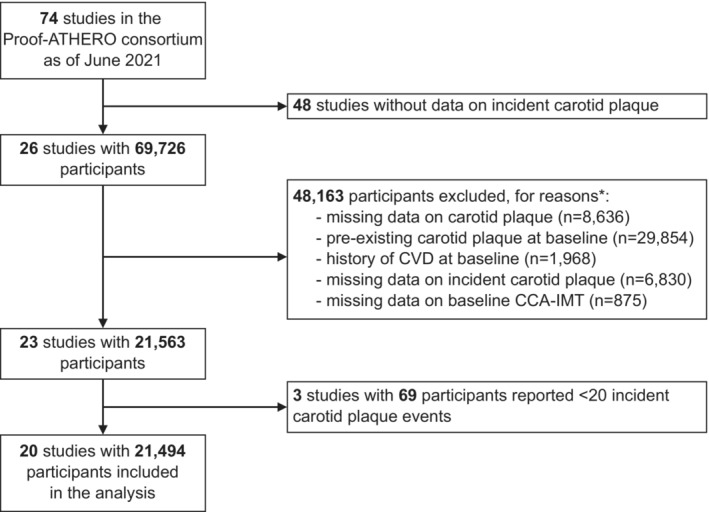
Flow diagram. *Exclusions were made hierarchically; 3 studies were omitted at this step because all participants of these studies had to be excluded. CCA‐IMT indicates common carotid artery intima‐media thickness; CVD, cardiovascular disease; and Proof‐ATHERO, Prospective Studies of Atherosclerosis.

The derivation of the study sample contributing to the present study is outlined in Figure 1. Of the 74 studies involved in the Proof‐ATHERO consortium, we excluded 48 that did not record incident carotid plaque. After further excluding participants who did not meet the prespecified inclusion criteria and excluding studies that recorded <20 incident carotid plaque events, a total of 20 studies involving 21 494 participants remained for analysis.^15^, ^41^, ^42^, ^43^, ^44^, ^45^, ^46^, ^47^, ^48^, ^49^, ^50^, ^51^, ^52^, ^53^, ^54^, ^55^, ^56^, ^57^, ^58^, ^59^

Table 1 and Table S3 summarize key characteristics of the studies and participants we analyzed. Twelve studies recruited participants from the general population, 6 recruited participants from high‐risk populations (ie, individuals with baseline coronary atherosclerosis, renal disease, or other vascular risk factors), and 2 were clinical trials (involving individuals on hemodialysis and with heterozygous familial hypercholesterolemia). The pooled mean age at baseline was 56 years (SD, 9 years); 55% of the participants were women. The overall mean of baseline CCA‐IMT values was 0.71 mm (SD, 0.17 mm), with 15 studies reporting mean CCA‐IMT values and 5 studies reporting maximum CCA‐IMT values. Over a median follow‐up of 5.9 years (5th–95th percentile, 1.9–19.0 years), 8278 participants (39%) developed first‐ever carotid plaque.

**Table 1:** Characteristics of Studies Contributing to the Analysis

Study acronym or first author	Total No.	Women, n (%)	Age, mean (SD), y	CCA‐IMT, mean (SD), mm	CCA‐IMT metric	Carotid plaque at any follow‐up	Focal plaque	Length of follow‐up, median (5th–95th percentile) y
General population								
AIR^41^	206	0 (0)	58 (1)	0.78 (0.12)	Mean	126	●	8.8 (3.1– 9.1)
ARIC^42^	7684	4572 (60)	53 (6)	0.61 (0.13)	Mean	2734	●	6.0 (2.8–23.7)
CHS^43^	917	650 (71)	71 (5)	0.93 (0.14)	Maximum	774	●	3.0 (2.8– 9.0)
CMCS‐BEIJING^44^	741	425 (57)	58 (8)	0.68 (0.21)	Mean	323	●	5.4 (5.4–5.5)
EVA^15^	769	485 (63)	65 (3)	0.65 (0.10)	Mean	116	●	3.9 (2.0–4.1)
KIHD^45^	552	0 (0)	49 (6)	0.71 (0.13)	Mean	313	●	18.0 (10.6–20.9)
MESA^46^	2101	1167 (56)	58 (9)	0.81 (0.16)	Maximum	1090	●	9.4 (8.8–10.4)
NOMAS‐INVEST^47^	278	169 (61)	66 (8)	0.70 (0.08)	Mean	125	●	5.6 (2.9–8.1)
PIVUS^48^	240	138 (58)	70 (0)	0.87 (0.14)	Mean	152	●	5.1 (5.0–5.3)
PLIC^49^	1315	805 (61)	54 (11)	0.63 (0.13)	Mean	303	●	6.0 (2.1–8.2)
ROTTERDAM^50^	1221	806 (66)	64 (6)	0.71 (0.11)	Mean	579	●	6.4 (6.1–7.2)
SAPHIR^51^	917	356 (39)	52 (6)	0.74 (0.11)	Mean	261	●	4.4 (4.1–6.2)
High‐risk populations								
BK REGISTRY^52^	213	82 (38)	58 (9)	0.78 (0.15)	Mean	32	●	1.4 (0.6–6.8)
CSN^53^	1713	743 (43)	54 (9)	0.93 (0.14)	Maximum	597	○	3.8 (1.2–10.7)
IMPROVE^54^	1107	711 (64)	63 (5)	0.70 (0.08)	Mean	387	○	2.5 (1.2–2.6)
Kato^55^	97	29 (30)	65 (13)	0.64 (0.13)	Mean	66	○	1.1 (0.9–1.7)
Landecho^56^	198	21 (11)	53 (9)	0.69 (0.14)	Maximum	63	●	3.6 (1.2–8.0)
NIGUARDA‐MONZINO^57^	498	233 (47)	49 (12)	0.79 (0.16)	Maximum	165	○	3.8 (1.2–9.1)
Clinical trials								
EGE STUDY^58^	117	70 (60)	54 (14)	0.71 (0.18)	Mean	23	NR	3.0 (3.0–3.0)
ENHANCE^59^	610	294 (48)	46 (9)	0.66 (0.14)	Mean	49	○	2.0 (0.5–2.1)
Total	21 494	11 756 (55)	56 (9)	0.71 (0.17)		8278		5.9 (1.9–19.0)

Full study names have been published previously.^33^ ● indicates “Yes” and ○ indicates “No”; CCA indicates common carotid artery; IMT, intima‐media thickness; and NR, not reported.

### Relationship Between CCA‐IMT and Development of Carotid Plaque

**Figure 2:**
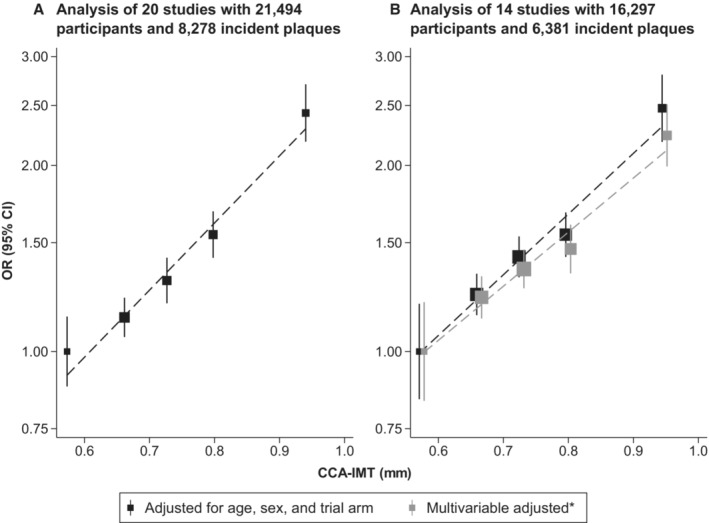
Odds ratios (ORs) for incident carotid plaque across quintiles of baseline common carotid artery intima‐media thickness (CCA‐IMT) in participants with complete data on age, sex, and trial arm (A) and variables used for multivariable adjustment* (B). The dashed lines indicate the best‐fitting lines through the odds ratio estimates. *Adjusted for age at baseline, sex, trial arm, ethnicity, smoking status at baseline, history of diabetes at baseline, systolic blood pressure at baseline, body mass index at baseline, low‐density lipoprotein cholesterol at baseline, high‐density lipoprotein cholesterol at baseline, intake of lipid‐lowering medication at baseline, and intake of antihypertensive medication at baseline.

Figure 2 depicts development of carotid plaque across quintiles of baseline CCA‐IMT. In the first, second, third, fourth, and fifth quintile, 1293 (28.9%), 1419 (33.1%), 1614 (36.8%), 1737 (41.7%), and 2215 (53.0%) individuals developed incident carotid plaque, respectively. The odds appeared to increase log‐linearly across CCA‐IMT quintiles when adjusting for age, sex, and trial arm as well as in the multivariable‐adjusted model.

**Figure 3:**
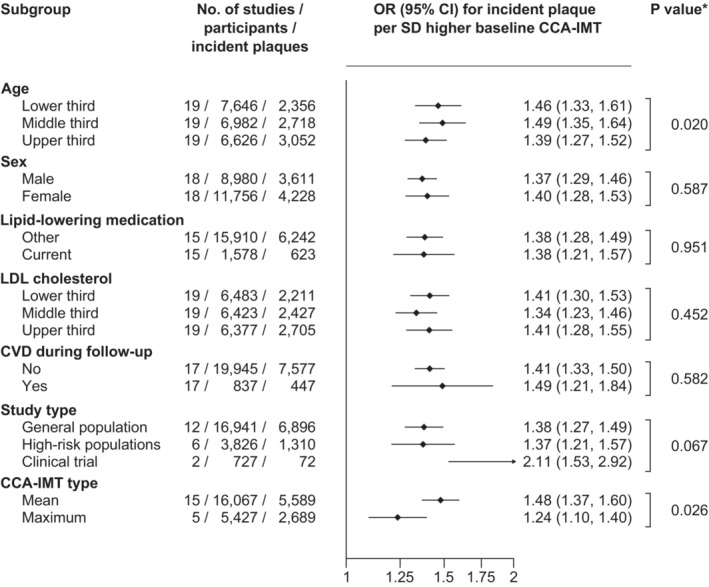
Comparison of the strength of association between baseline common carotid artery intima‐media thickness (CCA‐IMT) and incident carotid plaque across various subgroups. The models are additionally adjusted for age, sex, and trial arm, if appropriate. *P values from interaction for categorical participant‐level variables (ie, sex, lipid‐lowering medication, and cardiovascular disease [CVD] during follow‐up) and continuous participant‐level variables (ie, age and low‐density lipoprotein [LDL] cholesterol) and *P* values from meta‐regression for study‐level variables (ie, study type and CCA‐IMT type). After correcting for multiple testing, *P*≤0.0071 (0.05/7) was deemed statistically significant. Participant‐level subgroup analyses include only studies that contribute data to all levels of a subgroup. OR indicates odds ratio.

The pooled OR for first‐ever carotid plaque development, adjusted for age, sex, and trial arm, was 1.40 (95% CI, 1.31–1.50; *I* ^2^=63.9%) per SD higher level of baseline CCA‐IMT (for study‐specific estimates, see Figure S2). The corresponding OR per 0.1‐mm higher baseline level of CCA‐IMT was 1.30 (95% CI, 1.23–1.38; *I* ^2^=71.8%). As shown in Table 2, the association was slightly weakened when the OR was further adjusted for potential confounding variables. In a model further adjusted for ethnicity, smoking, diabetes, body mass index, systolic blood pressure, low‐ and high‐density lipoprotein cholesterol, and lipid‐lowering and antihypertensive medication, the OR per SD higher baseline CCA‐IMT was 1.34 (95% CI, 1.24–1.45; *I* ^2^=59.4%; 14 studies; 16 297 participants; 6381 incident carotid plaques). The ORs were virtually identical when further adjusted for estimated glomerular filtration rate or log‐transformed hs‐CRP values. In subgroup analyses (Figure 3), there was no evidence for effect modification by age, sex, intake of lipid‐lowering medication, low‐density lipoprotein cholesterol, development of CVD during follow‐up, type of study, and type of CCA‐IMT measure, when we considered a multiplicity‐adjusted threshold for statistical significance (all *P*>0.0071). In addition, we found no statistically significant difference in ORs by median duration of follow‐up, as demonstrated in Figure S3 (*P*=0.804). As shown in Table S4, results were also similar in sex‐specific analyses. The age‐ and trial arm–adjusted OR for incident carotid plaque per SD higher baseline CCA‐IMT was 1.38 (95% CI, 1.24–1.53; *I* ^2^=69.0%; 18 studies; 11 756 participants; 4228 incident carotid plaques) in women and 1.39 (95% CI, 1.31–1.46; *I* ^2^=10.8%; 18 studies; 8980 participants; 3611 incident carotid plaques) in men.

**Table 2:** Association Between Baseline CCA‐IMT and Incident Carotid Plaque Progressively Adjusted for Traditional and Emerging Cardiovascular Risk Factors

Level of adjustment	OR (95% CI) for incident carotid plaque per SD higher baseline CCA‐IMT	*P* value (*χ* ^2^)	*I* ^2^ (95% CI), %
Primary analysis	20 Studies; 21 494 participants; 8278 incident plaques		
Adjusted for age, sex, and trial arm	1.40 (1.31–1.50)	<0.001 (102.4)	63.9 (41.8–77.6)
Progressive adjustment*	14 Studies; 16 297 participants; 6381 incident plaques		
Adjusted for age, sex, and trial arm	1.40 (1.29–1.51)	<0.001 (66.2)	65.8 (39.9–80.6)
Above+ethnicity	1.40 (1.29–1.52)	<0.001 (65.8)	66.1 (40.4–80.7)
Above+smoking status	1.39 (1.28–1.51)	<0.001 (61.9)	66.2 (40.6–80.8)
Above+history of diabetes	1.38 (1.28–1.50)	<0.001 (60.6)	65.8 (39.9–80.6)
Above+body mass index	1.39 (1.28–1.51)	<0.001 (61.3)	65.2 (38.6–80.3)
Above+systolic blood pressure	1.36 (1.26–1.47)	<0.001 (60.1)	60.6 (29.4–78.1)
Above+LDL cholesterol	1.35 (1.25–1.46)	<0.001 (56.5)	59.7 (27.5–77.6)
Above+HDL cholesterol	1.34 (1.24–1.45)	<0.001 (56.2)	58.9 (25.8–77.2)
Above+lipid‐lowering medication	1.34 (1.24–1.45)	<0.001 (55.5)	59.4 (26.9–77.5)
Above+antihypertensive medication	1.34 (1.24–1.45)	<0.001 (55.0)	59.4 (26.8–77.4)
Further adjustment for eGFR*	10 Studies; 12 487 participants; 5274 incident plaques		
Multivariable adjusted†	1.30 (1.17–1.44)	<0.001 (25.4)	61.6 (23.5–80.7)
Above+eGFR	1.30 (1.17–1.44)	<0.001 (23.5)	63.4 (27.5–81.5)
Further adjustment for hs‐CRP*	12 Studies; 6987 participants; 2636 incident plaques		
Multivariable adjusted†	1.39 (1.30–1.48)	<0.001 (106.3)	0.0 (0.0–58.3)
Above+log hs‐CRP	1.39 (1.30–1.47)	<0.001 (104.3)	0.0 (0.0–58.3)

CCA indicates common carotid artery; eGFR, estimated glomerular filtration rate; HDL, high‐density lipoprotein; hs‐CRP, high‐sensitivity C‐reactive protein; IMT, intima‐media thickness; LDL, low‐density lipoprotein; and OR, odds ratio.

* Restricted to individuals having information on all variables included in the model.

^†^ Adjusted for age at baseline, sex, trial arm, ethnicity, smoking status at baseline, history of diabetes at baseline, systolic blood pressure at baseline, body mass index at baseline, low‐density lipoprotein cholesterol at baseline, high‐density lipoprotein cholesterol at baseline, intake of lipid‐lowering medication at baseline, and intake of antihypertensive treatment at baseline.

### Sensitivity Analyses

**Figure 4:**
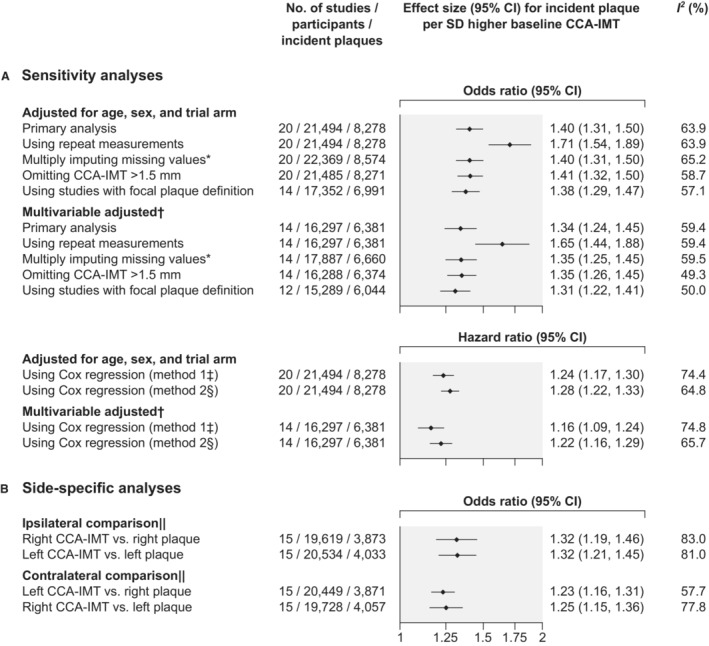
Sensitivity analyses (A) and side‐specific analyses (B) of the association between baseline common carotid artery intima‐media thickness (CCA‐IMT) and incident carotid plaque. Odds ratios were obtained from logistic regression analysis, and hazard ratios were obtained from Cox regression analysis. *Imputed variables (percentage of missing values that were imputed): CCA‐IMT (3.9%), ethnicity (0.1%), smoking status (3.4%), history of diabetes (4.5%), systolic blood pressure (1.5%), body mass index (1.1%), low‐density lipoprotein cholesterol (4.8%), high‐density lipoprotein cholesterol (3.6%), lipid‐lowering medication (2.2%), and antihypertensive medication (1.7%). ^†^Adjusted for age at baseline, sex, trial arm, ethnicity, smoking status at baseline, history of diabetes at baseline, systolic blood pressure at baseline, body mass index at baseline, low‐density lipoprotein cholesterol at baseline, high‐density lipoprotein cholesterol at baseline, intake of lipid‐lowering medication at baseline, and intake of antihypertensive medication at baseline. ^‡^In this model, date of carotid plaque development was estimated as the visit at which carotid plaque had first been detected. ^§^In this model, date of carotid plaque development was estimated as the midpoint between the visit at which carotid plaque had first been detected and the preceding visit. ^||^Adjusted for age at baseline, sex, and trial arm.

In sensitivity analyses, we observed similar ORs when we multiplied imputed missing values, excluded individuals with CCA‐IMT values >1.5 mm, or restricted analyses to studies that defined carotid plaque as focal thickening (Figure 4A). Stronger associations were observed when we considered long‐term averages (“usual levels”) of CCA‐IMT values, which we estimated on the basis of repeated CCA‐IMT measurements taken at a median of 2 occasions (range, 2–9 occasions). Median time between 2 consecutive CCA‐IMT measurements was 3.0 years (interquartile range, 2.3–5.4 years). The OR per SD higher “usual” CCA‐IMT was 1.71 (95% CI, 1.54–1.89; *I* ^2^=63.9%) when adjusted for age, sex, and trial arm and 1.65 (95% CI, 1.44–1.88; *I* ^2^=59.4%) in the multivariable‐adjusted model. When we used Cox regression and estimated the dates of plaque development as the visit at which plaque had first been detected, the hazard ratio (HR) for incident plaque per SD higher baseline CCA‐IMT was 1.24 (95% CI, 1.17–1.30; *I* ^2^=74.4%) when adjusted for age, sex, and trial arm and 1.16 (95% CI, 1.09–1.24; *I* ^2^=74.8%) in the multivariable‐adjusted model. When we estimated dates of plaque development as the midpoint between the visit at which plaque had first been detected and the preceding visit, HRs for incident plaque per SD higher baseline CCA‐IMT were 1.28 (95% CI, 1.22–1.33; *I* ^2^=64.8%) when adjusted for age, sex, and trial arm and 1.22 (95% CI, 1.16–1.29; *I* ^2^=65.7%) in the multivariable‐adjusted model. Finally, side‐specific analyses revealed somewhat stronger associations for an ipsilateral development than a contralateral development of carotid plaque (Figure 4B).

### Combined Meta‐Analysis With Aggregated Data

We identified 5 studies from the literature to supplement our multivariable individual participant data meta‐analysis (Figure S4).^14^, ^17^, ^27^, ^28^, ^29^ The pooled OR for carotid plaque per SD higher baseline CCA‐IMT based on data from these 5 studies was 1.28 (95% CI, 1.14–1.43; *I* ^2^=20.1%; 5 studies; 3736 participants). When meta‐analyzing ORs of the studies from the Proof‐ATHERO consortium that were included in the multivariable‐adjusted meta‐analysis with aggregated data of these 5 studies, the pooled OR for incident carotid plaque per SD higher baseline CCA‐IMT was 1.33 (95% CI, 1.24–1.42; *I* ^2^=54.1%; 18 studies; 19 295 participants).

## DISCUSSION

In the present individual participant data meta‐analysis embedded in the Proof‐ATHERO consortium, we investigated the association of CCA‐IMT values with the development of incident first‐ever carotid plaque during follow‐up. We observed an OR for plaque development of 1.40 (95% CI, 1.31–1.50) per SD higher level of baseline CCA‐IMT, which was reduced slightly in a multivariable adjustment model. We also demonstrated that odds increased approximately log‐linearly across quintiles of baseline CCA‐IMT. Finally, associations were robust in several sensitivity analyses and across a range of clinically relevant participant characteristics (eg, traditional risk factors and intake of medication) and study methods (eg, in assessing CCA‐IMT).

### Comparison With Previous Findings

We have previously investigated the relationship between CCA‐IMT and development of carotid plaque in a literature‐based meta‐analysis that considered 7 general population studies with a total of 9341 participants and 1288 events of carotid plaque.^30^ In this analysis preceding the current study, we had observed a pooled relative risk for incident carotid plaque of 1.78 (95% CI, 1.53–2.07), when comparing individuals in the top quartile of baseline CCA‐IMT with individuals in the bottom quartile. Although this effect size is comparable to the effect size in the current study (see results across quintiles in Figure 2), a key strength of the current analysis is that it included 6 times more incident outcomes and could therefore quantify the association more precisely (in addition to other advantages related to the individual participant data access). We were also able to include hitherto unpublished findings from 15 studies and extended the analysis to high‐risk populations and clinical trials. When meta‐analyzing the studies contributing to the Proof‐ATHERO consortium with the aggregated data of the additional studies we found in the literature,^14^, ^17^, ^27^, ^28^, ^29^ we identified a multivariable‐adjusted OR for incident carotid plaque of 1.33 (95% CI, 1.24–1.42; *I* ^2^=54.1%) per SD higher CCA‐IMT, which is nearly the same result as in the present multivariable‐adjusted primary analysis. Our findings are also in line with results from other studies in the literature that analyzed the association of CCA‐IMT with carotid plaque differently. The Tromsø study, for instance, observed a positive association between baseline cIMT and a higher number of plaques at follow‐up.^19^ The SHIP (Study of Health in Pomerania) reported that individuals with elevated CCA‐IMT had a higher risk for developing additional plaques in previously unaffected arterial segments.^18^ In contrast to these studies and our report, the Reykjavik Risk Evaluation for Infarct Estimates study found no statistically significant association between CCA‐IMT and formation of a new plaque.^20^

### Ultrasound Methods Used in the Contributing Studies

Measurement of cIMT and carotid plaque is generally performed noninvasively with high‐resolution B‐mode ultrasound. cIMT is defined as the so‐called double‐line pattern, representing the distance between the lumen‐intima and the media‐adventitia interfaces.^60^ The 2011 Mannheim cIMT and plaque consensus recommends cIMT to be measured at the far wall of the CCA in an area free of carotid plaque.^61^ In 2008, the American Society of Echocardiography also recommended measuring CCA‐IMT at the far wall of the carotid artery in their Consensus Statement but, contrarily, to include sections with carotid plaque.^62^ In the studies contributing to the present report, there were some differences in how CCA‐IMT was assessed (Table S2 and Figure S1). cIMT was often measured at different sections of the CCA, at the left and/or right side of the neck, and at the near and/or far wall of the CCA. To reduce variability and include a broad range of information from the entire CCA, we averaged all the available measurements to obtain an overall CCA‐IMT value. Moreover, in a meta‐regression analysis (Figure 3), we observed that the association was similarly strong in studies reporting mean CCA‐IMT and studies reporting maximum CCA‐IMT.

Besides different definitions of CCA‐IMT, studies also varied in terms of carotid plaque assessment (Table S2). The Mannheim cIMT and plaque consensus defines carotid plaque as focal thickening of at least 0.5 mm or 50% of its surrounding area or as cIMT >1.5 mm.^61^ Similarly, the American Society of Echocardiography recommends defining carotid plaque as “(1) any focal thickening thought to be atherosclerotic in origin and encroaching into the lumen of any segment of the carotid artery (protuberant‐type plaque) or (2) in the case of diffuse vessel wall atherosclerosis, when carotid intima‐media thickness measures ≥1.5 mm in any segment of the carotid artery (diffuse‐type plaque).”^63^ Although most of the studies contributing to our analysis defined carotid plaque as focal structure, some others defined it as cIMT above a predefined threshold. The latter may be problematic in the present analysis because cIMT is assumed to thicken progressively over time, and a direct association between elevated baseline cIMT and carotid plaque development in those studies would therefore be a logical consequence. Reassuringly, though, when we excluded these studies in a sensitivity analysis, the effect size pooled across the remaining studies was similar as in the primary analysis (OR, 1.38 [95% CI, 1.29–1.47]). Another potential challenge is that early stage of plaque development may sometimes be misclassified as elevated cIMT.^61^ Therefore, we conducted a sensitivity analysis that omitted individuals with CCA‐IMT >1.5 mm, which could be indicative for carotid plaque. Again, this analysis yielded results comparable to the primary analysis, with an overall OR for carotid plaque development of 1.41 (95% CI, 1.32–1.50) per SD higher level of baseline CCA‐IMT. Although we did not observe significant effect modification by differences in ultrasound protocols, discrepancies in definitions of cIMT and carotid plaque are suboptimal and standardizations of measurement techniques would be an essential approach to obtain adequate comparisons.^61^

### Clinical Implications

As atherosclerosis often develops over years without symptoms or detection, early identification of vulnerable individuals is the key to prevent its clinical sequelae. Current evidence shows that increased cIMT relates to unfavorable levels of risk factors,^64^, ^65^, ^66^ presence of atherosclerosis elsewhere in the arterial system,^4^ and the risk of future CVD events.^6^, ^7^ We have previously shown in an analysis of 119 clinical trials that different types of interventions reduce progression of cIMT and that the greater reductions in cIMT progression are associated with greater reductions in CVD risk, endorsing its usefulness as a surrogate marker.^67^ Leading on from this, we now provide further evidence for the role of cIMT as a risk marker for atherosclerotic disease, which may help to identify individuals at risk of developing advanced atherosclerotic lesions earlier.

### Strengths and Limitations

The present analysis has several strengths. First, we analyzed data of the Proof‐ATHERO consortium, the worldwide largest consortium with data on repeated assessments of atherosclerosis and CVD, and included 20 different studies with >21 000 individuals. Thus, a major strength of the current analysis is its large sample size, which allows estimating effect sizes with adequate precision. Second, we included data from studies in a variety of clinical settings, thereby enhancing the generalizability of our findings to various populations. Third, we excluded individuals with a history of CVD, reducing the potential influence of subsequent drug treatments or frequent medical checks on the development of carotid plaques. Fourth, access to individual participant data allowed us to harmonize outcomes, exposures, and levels of adjustment, and perform various participant‐level sensitivity analyses. Fifth, in a sensitivity analysis, we capitalized on the serial CCA‐IMT measurements available in our studies and estimated ORs for incident carotid plaque based on long‐term averages of CCA‐IMT rather than a single baseline measurement, thereby taking into account within‐person variation of CCA‐IMT during follow‐up. Sixth, because we had access to participant‐level data, we were able to study the shape of association between CCA‐IMT and development of carotid plaque across fifths of baseline CCA‐IMT. Seventh, compared with our previous literature‐based meta‐analysis, individual participant data meta‐analysis enabled a sophisticated analysis of effect sizes across participant‐level subgroups. Our study also has limitations. First, there were differences in how the individual studies defined and measured CCA‐IMT and carotid plaque. Second, because carotid plaque status was only available at the study visits and not in between, there was uncertainty about the exact time point of plaque development. For this reason, we prespecified to use logistic regression in our primary analysis. When we used Cox regression based on the estimated time to plaque development, HRs were highly significant, although numerically lower than ORs, as expected when the rare disease assumption is not met (39% developed the outcome plaque). Third, because the present meta‐analysis focused on plaque status, we cannot draw any conclusions about the relationship of CCA‐IMT with plaque quality, size, or architecture, which are more detailed measures to quantify and characterize carotid plaque.^8^ Fourth, our analysis includes long‐term follow‐up studies, with baseline examinations typically taking place in 1990s to early 2000s,^33^ and ultrasound devices have improved significantly since then. Consequently, it can be assumed that it would nowadays be possible to obtain ultrasound images with higher resolution, which would also enable us to identify plaques of smaller dimension. Furthermore, although recent guidelines also suggest measuring 3‐dimensional carotid plaque by applying modern ultrasound techniques,^63^ we were only able to analyze 2‐dimensional carotid plaque data because of the unavailability in our long‐term follow‐up studies. Also, we only investigated ultrasound‐based markers measured in the carotid arteries. Further research is needed on whether our results also hold for other vascular beds (eg, the femoral arteries).^8^ Finally, plaque data on specific carotid arterial segments (ie, CCA, carotid bifurcation, and internal carotid artery) were sparse and could not be considered in our analysis, which prevented us from investigating the association between CCA‐IMT and segment‐specific development of carotid plaque.

## CONCLUSIONS

In this large‐scale meta‐analysis based on participant‐level data, CCA‐IMT was associated with the long‐term risk of developing first‐ever carotid plaque, independent of traditional cardiovascular risk factors. The association was robust across sensitivity analyses and similarly strong for women and men and for individuals at different ages.

## Sources of Funding

This research was funded in whole, or in part, by the Austrian Science Fund (FWF) (grant P 32488). For the purpose of open access, the author has applied a CC BY public copyright license to any author accepted manuscript version arising from this submission.

## Disclosures

Prof Rundek reports grants from National Institutes of Health/National Institute of Neurological Disorders and Stroke and holds a leadership or fiduciary role in the Intersocietal Accreditation Commission. Dr Gerstein reports grants from Sanofi, Eli Lilly, AstraZeneca, Boehringer Ingelheim, Novo Nordisk, Merck, Abbott, and Hanmi; and personal fees from Sanofi, Eli Lilly, AstraZeneca, Boehringer Ingelheim, Abbott, Novo Nordisk, Kowa Research Institute, Zuellig, and DKSH outside the submitted work. Prof Olsen reports honoraria from Novo Nordisk A/S, AstraZeneca, and Boehringer Ingelheim outside the submitted work. Prof Sattar reports grants or contracts from AstraZeneca, Boehringer Ingelheim, Novartis, and Roche Diagnostics; consulting fees from Abbott Laboratories, Afimmune, Amgen, AstraZeneca, Boehringer Ingelheim, Eli Lilly, Hanmi Pharmaceuticals, Merck Sharp & Dohme, Novartis, Novo Nordisk, Pfizer, Roche Diagnostics, and Sanofi; and payment or honoraria from Abbott Laboratories, AstraZeneca, Boehringer Ingelheim, Eli Lilly, Janssen, and Novo Nordisk outside the submitted work. Dr Landecho reports payment or honoraria and receipt of equipment from NovoNordisk. Prof Norata reports grants or contracts from Novartis; and payment or honoraria from Novartis outside the submitted work. Prof Catapano reports payment or honoraria from Aegerion, Akcea, Amarin, Amgen, Amryt, Astrazeneca, Daiichi Sankyo, Esperion, Ionis Pharmaceuticals, Kowa, Medscape, Menarini, Merck, Mylan, Novartis, PeerVoice, Pfizer, Recordati, Regeneron, Sandoz, Sanofi, and The Corpus outside the submitted work. Dr Sweeting is a full‐time employee of AstraZeneca; holds shares in AstraZeneca plc; and reports a grant from the German Research Foundation. The remaining authors have no disclosures to report.

## Supporting information

Appendix List of Proof‐ATHERO study group members
Tables S1–S4
Figures S1–S4
