# Supplementary material for: Association of Intima‐Media Thickness Measured at the Common Carotid Artery With Incident Carotid Plaque: Individual Participant Data Meta‐Analysis of 20 Prospective Studies
Source: J Am Heart Assoc. 2023 Jun 10;12(12):e027657. doi: 10.1161/JAHA.122.027657 (PMC10356054; doi:10.1161/JAHA.122.027657)
Supplement: Supplementary file 1 — Appendix List of Proof‐ATHERO study group members Tables S1–S4 Figures S1–S4 [file JAH3-12-e027657-s001.pdf]

SUPPLEMENTAL MATERIAL

Appendix. List of Proof-ATHERO study group members

Stefan Agewall, MD, PhD
Department of Clinical Sciences, Danderyd Hospital division of Cardiology
Karolinska Institutet
Stockholm, Sweden
and
Institute of Clinical Sciences
University of Oslo
Oslo, Norway

Tadao Akizawa, MD, PhD
Division of Nephrology, Department of Medicine
Showa University School of Medicine
Tokyo, Japan

Mayuko Amaha, MD
Division of Nephrology
Shinmatsudo Central General Hospital
Chiba, Japan

Mauro Amato, PhD
Centro Cardiologico Monzino IRCCS
Milan, Italy

Aleksandra Araszkiewicz, MD, PhD
Department of Internal Medicine and Diabetology
Poznan University of Medical Sciences
Poznan, Poland

Gülay Aşçi, MD
Nephrology Department
Ege University School of Medicine
Bornova-Izmir, Turkey

Folkert W. Asselbergs, MD, PhD
Department of Cardiology
University Medical Center Utrecht
Utrecht, the Netherlands

Jang-Ho Bae, MD, FACC
Heart Center
Konyang University Hospital
Daejeon, South Korea
and
Department of Cardiology
Konyang University College of Medicine
Daejeon, South Korea

Tatyana Balakhonova, MD, PhD
Ultrasound Vascular Laboratory
National Medical Research Center of Cardiology
Moscow, Russia

Damiano Baldassarre, PhD
Department of Medical Biotechnology and Translational Medicine
University of Milan
Milan, Italy
and
Centro Cardiologico Monzino IRCCS
Milan, Italy

Edith Beishuizen, MD
Department of Internal Medicine
HMC+ (Bronovo)
the Hague, the Netherlands

Oscar Beloqui, MD, PhD
Department of Internal Medicine
University Clinic of Navarra
Navarra, Spain

Göran Bergström, MD, PhD
Department of Molecular and Clinical Medicine, Institute of Medicine, Sahlgrenska Academy
University of Gothenburg
Gothenburg, Sweden
and
Department of Clinical Physiology
Sahlgrenska University Hospital, Region Västragötaland
Gothenburg, Sweden

Enrique Bernal, MD, PhD
Infectious Diseases Unit
Reina Sofia Hospital
Murcia, Spain

Joline W. J. Beulens, PhD
Department of Epidemiology & Data Science
Amsterdam UMC- Location Vumc
Amsterdam, the Netherlands

Sebastjan Bevc, MD, PhD
Department of Nephrology
University Medical Centre Maribor
Maribor, Slovenia
and
Faculty of Medicine
University of Maribor
Maribor, Slovenia

Lokpal Bhatia, MD, MRCP, PhD
Faculty of Medicine
University of Southampton – Southampton General Hospital
Southampton, UK
and
Southampton NIHR Biomedical Research Centre
University Hospital Southampton – Southampton General Hospital
Southampton, UK

Horst Bickel, PhD
Department of Psychiatry and Psychotherapy
Technische Universität München
Munich, Germany

Peter J. Blankestijn, MD
Department of Nephrology
University Medical Center Utrecht
Utrecht, the Netherlands

Lena Bokemark, MD, PhD
Wallenberg Laboratory for Cardiovascular Research
University of Gothenburg
Gothenburg, Sweden

Michiel L. Bots, MD, PhD
Julius Center for Health Sciences and Primary Care
University Medical Center Utrecht
Utrecht, the Netherlands

Frank P. Brouwers, MD, PhD
Department of Cardiology
Haga Teaching Hospital
the Hague, the Netherlands

Christopher D. Byrne, FRCP, FRCPath, PhD
School of Human Development and Health, Faculty of Medicine
University of Southampton
Southampton, UK
and
Southampton National Institute for Health and Care Research, Biomedical Research Centre
University Hospital Southampton
Southampton, UK

Laura Calabresi, PhD
Department of Pharmacological and Biomolecular Sciences
University of Milan
Milan, Italy

Philip C. Calder, PhD
School of Human Development and Health, Faculty of Medicine
University of Southampton
Southampton, UK
and
Southampton National Institute for Health and Care Research, Biomedical Research Centre
University Hospital Southampton
Southampton, UK

Samuela Castelnuovo, PhD
Centro Dislipidemie
ASST Grande Ospedale Metropolitano Niguarda
Milan, Italy

Alberico Catapano, PhD, MDhc
Department of Pharmacological and Biomolecular Sciences
University of Milan
Milan, Italy
and
IRCCS Multimedica
Milan, Italy

Pei-Chun Chen, PhD
Clinical Informatics & Medical Statistics Research Center
Chang Gung University
Taoyuan, Taiwan

Kuo-Liong Chien, MD, PhD
Institute of Epidemiology and Preventive Medicine
National Taiwan University
Taipei, Taiwan

Ana R. Cunha, PhD
Department of Clinical Medicine
State University of Rio de Janeiro
Rio de Janeiro, Brazil

Jesse Dawson, MD, FRCP, FESO
Institute of Cardiovascular and Medical Sciences
University of Glasgow
Glasgow, UK

Eric de Groot, MD, PhD
Imagelabonline & Cardiovascular
Erichem, the Netherlands
and
Department of Gastroenterology and Hepatology
Amsterdam UMC – Academic Medical Centre
Amsterdam, the Netherlands

Moise Desvarieux, MD, PhD
Department of Epidemiology
Mailman School of Public Health, Columbia University
New York, USA
and
METHODS Core, Centre de Recherche Epidémiologie et Statistique Paris Sorbonne Cité (CRESS)
Institut National de la Santé et de la Recherche Médicale (INSERM) UMR 1153
Paris, France

Chrysostomos Dimitriadis, MD
University Department of Nephrology
Hippokration General Hospital
Thessaloniki, Greece

Pierre Ducimetiere, PhD
Faculty of Medicine
University Paris Descartes
Paris, France

Robert Ekart, MD, PhD
Department of Dialysis
University Medical Centre Maribor
Maribor, Slovenia

Petra Elders, MD, PhD
Department of General Practice
Amsterdam UMC- Location Vumc
Amsterdam, the Netherlands

Jean Philippe Empana, MD, PhD
Paris Cardiovascular Research Centre (PARCC)
University Paris Descartes
Paris, France

Mark A. Espeland, PhD
Department of Biostatistics and Data Science
Wake Forest School of Medicine
Winston-Salem, NC, USA

Marat Ezhov, MD, PhD
Laboratory of Lipid Disorders
National Medical Research Center of Cardiology
Moscow, Russia

Beat Frauchiger, MD
Department of Internal Medicine
Kantonsspital Frauenfeld
Frauenfeld, Switzerland

Alfonsa Friera, MD
Radiology Department
Universidad Autónoma de Madrid
Madrid, Spain

Rafael Gabriel, MD, PhD
National School of Public Health
Instituto de Salud Carlos III
Madrid, Spain

Hertzel C. Gerstein, MD, MSc, FRCPC
Department of Medicine and Population Health Research Institute
McMaster University
Hamilton, Ontario, Canada
and
Hamilton General Hospital
Hamilton, Ontario, Canada

Paolo Gresele, MD, PhD
Division of Internal and Cardiovascular Medicine,
Department of Medicine and Surgery
University of Perugia
Perugia, Italy

Liliana Grigore, MD, PhD
IRCCS Multimedica
Milan, Italy

Diederick E. Grobbee, MD, PhD
Julius Center for Health Sciences and Primary Care
University Medical Center Utrecht
Utrecht, the Netherlands

Muriel P. C. Grooteman, MD, PhD
Department of Nephrology,
Amsterdam Cardiovascular Sciences
Amsterdam UMC
Amsterdam, the Netherlands

Giuseppe Guglielmini, PhD
Division of Internal and Cardiovascular Medicine,
Department of Medicine and Surgery
University of Perugia
Perugia, Italy

Markolf Hanefeld, MD, DHC, PhD
Center for Clinical Studies
Technical University Dresden
Dresden, Germany

Peter Higgins, MD, MRCP
Institute of Cardiovascular and Medical Sciences
University of Glasgow
Glasgow, UK

Radovan Hojs, MD, PhD
Department of Nephrology
University Medical Centre Maribor
Maribor, Slovenia
and
Faculty of Medicine
University of Maribor
Maribor, Slovenia

Hirokazu Honda, MD, PhD
Division of Nephrology,
Department of Medicine
Showa University School of Medicine
Tokyo, Japan

Satoshi Hoshide, MD, PhD
Department of Medicine
Jichi Medical University School of Medicine
Tochigi, Japan

Menno V. Huisman, MD, PhD
Department of Thrombosis and Hemostasis
Leiden University Medical Center
Leiden, the Netherlands

Bernhard Iglseder, MD
Department of Geriatric Medicine
Gemeinnützige Salzburger Landeskliniken Betriebsgesellschaft GmbH
Christian-Doppler-Klinik
Salzburg, Austria
and
Department of Geriatric Medicine
Paracelsus Medical University
Salzburg, Austria

M. Arfan Ikram, MD, PhD
Department of Epidemiology
Erasmus University Medical Center
Rotterdam, the Netherlands

Raffaele Izzo, MD, PhD
Department of Advanced Biomedical Sciences
Federico II University
Naples, Italy

Lisa M. Jamieson, PhD
Australian Research Centre for Population Oral Health
University of Adelaide
Adelaide, SA, Australia

Aleksandar Jovanovic, MD, PhD
Faculty of Medicine
University of Prishtina
Prishtina\Kosovska Mitrovica, Serbia

Kostas Kapellas, PhD
Australian Research Centre for Population Oral Health
University of Adelaide
Adelaide, SA, Australia

Kazuomi Kario, MD, PhD
Department of Medicine
Jichi Medical University School of Medicine
Tochigi, Japan

John J. P. Kastelein, MD, PhD, FESC
Department of Vascular Medicine
Academic Medical Centre,
University of Amsterdam
Amsterdam, the Netherlands

Akihiko Kato, MD
Blood Purification Unit
Hamamatsu University
Hospital
Hamamatsu, Japan

Jussi Kauhanen, MD
Institute of Public Health and
Clinical Nutrition
University of Eastern Finland,
Kuopio Campus
Kuopio, Finland

Maryam Kavousi, MD, PhD,
FESC
Department of Epidemiology
Erasmus University Medical
Center
Rotterdam, the Netherlands

Stefan Kiechl, MD
Department of Neurology
Medical University of
Innsbruck
Innsbruck, Austria
and
VASCage GmbH
Research Centre on Vascular
Ageing and Stroke
Innsbruck, Austria

Kazuo Kitagawa, MD, PhD
Department of Neurology
Tokyo Women's Medical
University
Tokyo, Japan

Sverre E. Kjeldsen, MD, PhD
Department of Cardiology
Oslo University Hospital
Oslo, Norway

Gerhard Klingenschmid, MD
Department of Neurology
Medical University of
Innsbruck
Innsbruck, Austria

Manuel F. Landecho, MD, PhD
Department of Internal
Medicine
University Clinic of Navarra
Navarra, Spain

Tatjana Lazarevic, MA
Faculty of Medicine
University of Kragujevac
Kragujevac, Serbia

Moo-Sik Lee, MD, PhD
Department of Preventive
Medicine, College of Medicine
Konyang University
Daejeon, South Korea
and
Department of Occupational
and Environmental Medicine
Konyang University Hospital
Daejeon, South Korea

Hung-Ju Lin, MSc
Department of Internal
Medicine
National Taiwan University
Hospital
Taipei, Taiwan

Lars Lind, MD, PhD
Department of Medicine
Uppsala University
Uppsala, Sweden

Jing Liu, MD, PhD
Department of Epidemiology
Beijing Anzhen Hospital,
Capital Medical University
Beijing, China

Eva Lonn, MD, MSc, FRCPC,
FACC
Department of Medicine and
Population Health Research
Institute
McMaster University
Hamilton, Ontario, Canada
and
Hamilton General Hospital
Hamilton, Ontario, Canada

Matthias W. Lorenz, MD, PD
Department of Neurology
Goethe University
Frankfurt am Main, Germany
and
Klinik für Neurologie
Krankenhaus Nordwest
Frankfurt
Frankfurt am Main, Germany

Dianna Magliano, PhD
Department of Epidemiology
and Preventive Medicine
Monash University, Alfred
Hospital
Melbourne, Australia

Costantino Mancusi, MD
Department of Advanced
Biomedical Sciences
Federico II University
Naples, Italy

Maria V. Manzi, MD
Department of Advanced
Biomedical Sciences
Federico II University
Naples, Italy

Stela McLachlan, PhD
Usher Institute
University of Edinburgh
Edinburgh, UK

John McNeil, PhD, MBBS
School of Public Health and
Preventive Medicine
Monash University
Melbourne, Australia

Rino Migliacci, MD, PhD
Division of Internal Medicine
Cortona Hospital
Cortona, Italy

Michiaki Nagai, MD, PhD
Department of Internal
Medicine, General Medicine
and Cardiology
Hiroshima City Asa Hospital
Hiroshima, Japan

Tsukasa Nakamura, MD, PhD
Division of Nephrology
Shinmatsudo Central General
Hospital
Chiba, Japan

Prabath W. B. Nanayakkara,
MD, PhD, FRCP
Department of Clinical
Neurophysiology
Amsterdam UMC
Amsterdam, the Netherlands

Dariusz Naskręt, MD, PhD
Department of Internal
Medicine and Diabetology
Poznan University of Medical
Sciences
Poznan, Poland

Mario F. Neves, MD, PhD
Department of Clinical
Medicine
State University of Rio de
Janeiro
Rio de Janeiro, Brazil

Pythia T. Nieuwkerk, PhD
Department of Medical Psychology
Amsterdam UMC- Location AMC
Amsterdam, the Netherlands

Giuseppe D. Norata, PhD
SISA Center for the Study of Atherosclerosis
Bassini Hospital
Cinisello Balsamo, Italy
and
Department of Pharmacological and Biomolecular Sciences
University of Milan
Milan, Italy

Ercan Ok, MD
Nephrology Department
Ege University School of Medicine
Bornova-Izmir, Turkey

Shuhei Okazaki, MD, PhD
Department of Neurology
Osaka University Graduate School of Medicine
Osaka, Japan

Michael H. Olsen, MD, PhD, DMSc
Department of Internal Medicine, Holbaek Hospital
University of Southern Denmark
Odense, Denmark

Aikaterini Papagianni, MD, PhD
Department of Nephrology, School of Medicine
Aristotle University of Thessaloniki, General Hospital "Hippokratio"
Thessaloniki, Greece

Hyun-Woong Park, MD
Division of Cardiology, Department of Internal Medicine
Chungnam National University Sejong Hospital
Sejong-si, South Korea

Grace Parraga, PhD, FCAHS
Department of Medical Biophysics
Robarts Research Institute, Western University
London, ON, Canada

Matthieu Plichart, MD, PhD
Paris Cardiovascular Research Centre (PARCC)
University Paris Descartes
Paris, France
and
Fondation Santé Service Hospital at Home
Levallois-Perret, France

Holger Poppert, MD, PhD
Department of Neurology
Technische Universität München
Munich, Germany

David Preiss, PhD, FRCPath, MRCP
MRC Population Health Research Unit, Clinical Trial Service Unit, Nuffield Department of Population Health
University of Oxford
Oxford, UK

Jackie F. Price, MD
Usher Institute
University of Edinburgh
Edinburgh, UK

Peter Reiss, MD, PhD
Department of Global Health
Amsterdam UMC- Location AMC
Amsterdam, the Netherlands
and
Amsterdam Institute for Global Health and Development
University of Amsterdam
Amsterdam, the Netherlands

Tatjana Rundek, MD, PhD
Department of Neurology
University of Miami Miller School of Medicine
Miami, USA

Femke Rutters, PhD
Department of Epidemiology & Data Science
Amsterdam UMC- Location Vumc
Amsterdam, the Netherlands

Maya S. Safarova, MD, PhD
Department of Cardiovascular Medicine
University of Kansas Medical Center
Kansas City, KS, USA

Dirk Sander, MD
Department of Neurology
Benedictus Hospital Tutzing & Feldafing
Feldafing, Germany
and
Department of Neurology
Technische Universität München
Munich, Germany

Eiichi Sato, MD
Division of Nephrology
Shinmatsudo Central General Hospital
Chiba, Japan

Naveed Sattar, MD, PhD
BHF Glasgow Cardiovascular Research Centre
University of Glasgow
Glasgow, UK

Caroline Schmidt, PhD
Wallenberg Laboratory for Cardiovascular Research
University of Gothenburg
Gothenburg, Sweden

Lisa Seekircher, DI, Mag., BSc
Institute of Health Economics
Medical University of Innsbruck
Innsbruck, Austria

Matthias Sitzer, MD
Department of Neurology
Klinikum Herford
Herford, Germany
and
Department of Neurology
Goethe University
Frankfurt am Main, Germany

Michael Skilton, PhD
Charles Perkins Centre, Faculty of Medicine and Health
University of Sydney
Sydney, NSW, Australia

J. David Spence, CM, MD, FRCPC, FAHA
Stroke Prevention & Atherosclerosis Research Centre
Robarts Research Institute, Western University
London, ON, Canada

Daniel Staub, MD
Department of Angiology
University Hospital Basel
Basel, Switzerland

Coen D. A. Stehouwer, MD, PhD, FESC
Department of Internal Medicine and Cardiovascular Research Institute Maastricht (CARIM)
Maastricht University Medical Centre
Maastricht, the Netherlands

Helmuth Steinmetz, MD
Department of Neurology
Goethe University
Frankfurt am Main, Germany

Radojica Stolić, MD, PhD
Department of Internal Medicine, Faculty of Medical Sciences
University of Kragujevac
Kragujevac, Serbia

Erik Stroes, MD, PhD
Department of Vascular Medicine
Academic Medical Centre, University of Amsterdam
Amsterdam, the Netherlands

Ta-Chen Su, MD, PhD
Department of Internal Medicine
National Taiwan University Hospital
Taipei, Taiwan

Carmen Suarez, MD, PhD
Internal Medicine Department
Universidad Autónoma de Madrid
Madrid, Spain

Michael J. Sweeting, PhD
Department of Health Sciences
University of Leicester
Leicester, UK
and
Department of Public Health and Primary Care
University of Cambridge
Cambridge, UK

Pieter M. ter Wee, MD, PhD
Department of Nephrology
Amsterdam UMC
Amsterdam, the Netherlands

Elena Tremoli, PhD
Maria Cecilia Hospital
Cotignola (RA), Italy

Lena Tschiderer, DI, BSc, PhD
Institute of Health Economics
Medical University of Innsbruck
Innsbruck, Austria

Tomi-Pekka Tuomainen, MD, PhD
Institute of Public Health and Clinical Nutrition
University of Eastern Finland, Kuopio Campus
Kuopio, Finland

Aleksandra Uruska, MD, PhD
Department of Internal Medicine and Diabetology
Poznan University of Medical Sciences
Poznan, Poland

Heiko Uthoff, MD, PD
Department of Angiology
University Hospital Basel
Basel, Switzerland

Michiel A. van Agtmael, MD, PhD
Department of Internal Medicine
Amsterdam UMC, Vrije Universiteit
Amsterdam, the Netherlands

Wiek van Gilst, PhD
Department of Experimental Cardiology
University Medical Center Groningen
Groningen, the Netherlands

Marit G. A. van Vonderen, MD, PhD
Department of Internal Medicine
Medical Center Leeuwarden
Leeuwarden, the Netherlands

Fabrizio Veglia, PhD
Maria Cecilia Hospital
Cotignola (RA), Italy

Frank L. J. Visseren, MD
Department of Vascular Medicine
University Medical Center Utrecht
Utrecht, the Netherlands

Ari Voutilainen, PhD
Institute of Public Health and Clinical Nutrition
University of Eastern Finland, Kuopio Campus
Kuopio, Finland

Kristian Wachtell, MD, PhD, DrMedSci
Department of Cardiology
Oslo University Hospital
Oslo, Norway

Matthew Walters, MD, FRCP
School of Medicine, Dentistry and Nursing
University of Glasgow
Glasgow, UK

Miao Wang, MD
Department of Epidemiology
Beijing Anzhen Hospital, Capital Medical University
Beijing, China

Thapat Wannarong, MD
Department of Neurology, Neurological Institute
University Hospitals Cleveland Medical Center, Case Western Reserve University School of Medicine
Cleveland, OH, USA

Johann Willeit, MD
Department of Neurology
Medical University of Innsbruck
Innsbruck, Austria

Peter Willeit, MD, MPhil, PhD
Institute of Health Economics
Medical University of
Innsbruck
Innsbruck, Austria
and
Department of Public Health
and Primary Care
University of Cambridge
Cambridge, UK

Miles D. Witham, BMBCh,
PhD
AGE Research Group, NIHR
Newcastle Biomedical
Research Centre
Newcastle University and
Newcastle-upon-Tyne
Hospitals Trust
Newcastle, UK

Salim Yusuf, MD, DPhil,
MRCP
Department of Medicine and
Population Health Research
Institute
McMaster University
Hamilton, Ontario, Canada
and
Hamilton General Hospital
Hamilton, Ontario, Canada

Dong Zhao, MD, PhD
Department of Epidemiology
Beijing Anzhen Hospital,
Capital Medical University
Beijing, China

Zhi-Yong Zou, MD
Institute of Child and
Adolescent Health, School of
Public Health
Peking University
Beijing, China

Sophia Zoungas, MD, FRACP,
PhD
School of Public Health and
Preventive Medicine
Monash University
Melbourne, Australia

Dorota A. Zozulińska-
Ziółkiewicz, MD, PhD
Department of Internal
Medicine and Diabetology
Poznan University of Medical
Sciences
Poznan, Poland

Table S1. PRISMA-IPD Checklist.

PRISMA-IPD Section/topic	Item No	Checklist item	Reported on page
**Title**			
Title	1	Identify the report as a systematic review and meta-analysis of individual participant data.	1
**Abstract**			
Structured summary	2	Provide a structured summary including as applicable:	5-6
		**Background**: state research question and main objectives, with information on participants, interventions, comparators and outcomes.	
		**Methods**: report eligibility criteria; data sources including dates of last bibliographic search or elicitation, noting that IPD were sought; methods of assessing risk of bias.	
		**Results**: provide number and type of studies and participants identified and number (%) obtained; summary effect estimates for main outcomes (benefits and harms) with confidence intervals and measures of statistical heterogeneity. Describe the direction and size of summary effects in terms meaningful to those who would put findings into practice.	
		**Discussion:** state main strengths and limitations of the evidence, general interpretation of the results and any important implications.	
		**Other:** report primary funding source, registration number and registry name for the systematic review and IPD meta-analysis.	
**Introduction**			
Rationale	3	Describe the rationale for the review in the context of what is already known.	9
Objectives	4	Provide an explicit statement of the questions being addressed with reference, as applicable, to participants, interventions, comparisons, outcomes and study design (PICOS). Include any hypotheses that relate to particular types of participant-level subgroups.	10
**Methods**			
Protocol and registration	5	Indicate if a protocol exists and where it can be accessed. If available, provide registration information including registration number and registry name. Provide publication details, if applicable.	10
Eligibility criteria	6	Specify inclusion and exclusion criteria including those relating to participants, interventions, comparisons, outcomes, study design and characteristics (e.g. years when conducted, required minimum follow-up). Note whether these were applied at the study or individual level i.e. whether eligible participants were included (and ineligible participants excluded) from a study that included a wider population than specified by the review inclusion criteria. The rationale for criteria should be stated.	10-11
Identifying studies – information sources	7	Describe all methods of identifying published and unpublished studies including, as applicable: which bibliographic databases were searched with dates of coverage; details of any hand searching including of conference proceedings; use of study registers and agency or company databases; contact with the original research team and experts in the field; open adverts and surveys. Give the date of last search or elicitation.	10-11
Identifying studies – search	8	Present the full electronic search strategy for at least one database, including any limits used, such that it could be repeated.	11

Study selection processes	9	State the process for determining which studies were eligible for inclusion.	10-11
Data collection processes	10	Describe how IPD were requested, collected and managed, including any processes for querying and confirming data with investigators. If IPD were not sought from any eligible study, the reason for this should be stated (for each such study).<br>If applicable, describe how any studies for which IPD were not available were dealt with. This should include whether, how and what aggregate data were sought or extracted from study reports and publications (such as extracting data independently in duplicate) and any processes for obtaining and confirming these data with investigators.	10, doi: 10.1159/000508498
Data items	11	Describe how the information and variables to be collected were chosen. List and define all study level and participant level data that were sought, including baseline and follow-up information. If applicable, describe methods of 2tandardizing or translating variables within the IPD datasets to ensure common scales or measurements across studies.	10-13
IPD integrity	A1	Describe what aspects of IPD were subject to data checking (such as sequence generation, data consistency and completeness, baseline imbalance) and how this was done.	10-13
Risk of bias assessment in individual studies.	12	Describe methods used to assess risk of bias in the individual studies and whether this was applied separately for each outcome. If applicable, describe how findings of IPD checking were used to inform the assessment. Report if and how risk of bias assessment was used in any data synthesis.	11-13
Specification of outcomes and effect measures	13	State all treatment comparisons of interests. State all outcomes addressed and define them in detail. State whether they were pre-specified for the review and, if applicable, whether they were primary/main or secondary/additional outcomes. Give the principal measures of effect (such as risk ratio, hazard ratio, difference in means) used for each outcome.	11-12
Synthesis methods	14	Describe the meta-analysis methods used to synthesise IPD. Specify any statistical methods and models used. Issues should include (but are not restricted to):<br>• Use of a one-stage or two-stage approach.<br>• How effect estimates were generated separately within each study and combined across studies (where applicable).<br>• Specification of one-stage models (where applicable) including how clustering of patients within studies was accounted for.<br>• Use of fixed or random effects models and any other model assumptions, such as proportional hazards.<br>• How (summary) survival curves were generated (where applicable).<br>• Methods for quantifying statistical heterogeneity (such as I^2 and τ^2).<br>• How studies providing IPD and not providing IPD were analysed together (where applicable).<br>• How missing data within the IPD were dealt with (where applicable).	11-14
Exploration of variation in effects	A2	If applicable, describe any methods used to explore variation in effects by study or participant level characteristics (such as estimation of interactions between effect and covariates). State all participant-level characteristics that were analysed as potential effect modifiers, and whether these were pre-specified.	11-13
Risk of bias across studies	15	Specify any assessment of risk of bias relating to the accumulated body of evidence, including any pertaining to not obtaining IPD for particular studies, outcomes or other variables.	11-13
Additional analyses	16	Describe methods of any additional analyses, including sensitivity analyses. State which of these were pre-specified.	12-13

**Results**			
Study selection and IPD obtained	17	Give numbers of studies screened, assessed for eligibility, and included in the systematic review with reasons for exclusions at each stage. Indicate the number of studies and participants for which IPD were sought and for which IPD were obtained. For those studies where IPD were not available, give the numbers of studies and participants for which aggregate data were available. Report reasons for non-availability of IPD. Include a flow diagram.	Figure 1, Figure S4
Study characteristics	18	For each study, present information on key study and participant characteristics (such as description of interventions, numbers of participants, demographic data, unavailability of outcomes, funding source, and if applicable duration of follow-up). Provide (main) citations for each study. Where applicable, also report similar study characteristics for any studies not providing IPD.	Table 1, Table S2
IPD integrity	A3	Report any important issues identified in checking IPD or state that there were none.	10
Risk of bias within studies	19	Present data on risk of bias assessments. If applicable, describe whether data checking led to the up-weighting or down-weighting of these assessments. Consider how any potential bias impacts on the robustness of meta-analysis conclusions.	14-16
Results of individual studies	20	For each comparison and for each main outcome (benefit or harm), for each individual study report the number of eligible participants for which data were obtained and show simple summary data for each intervention group (including, where applicable, the number of events), effect estimates and confidence intervals. These may be tabulated or included on a forest plot.	Figure S2
Results of syntheses	21	Present summary effects for each meta-analysis undertaken, including confidence intervals and measures of statistical heterogeneity. State whether the analysis was pre-specified, and report the numbers of studies and participants and, where applicable, the number of events on which it is based.	15-17, Figure 2, Figure 3, Figure S2,
		When exploring variation in effects due to patient or study characteristics, present summary interaction estimates for each characteristic examined, including confidence intervals and measures of statistical heterogeneity. State whether the analysis was pre-specified. State whether any interaction is consistent across trials.	
		Provide a description of the direction and size of effect in terms meaningful to those who would put findings into practice.	
Risk of bias across studies	22	Present results of any assessment of risk of bias relating to the accumulated body of evidence, including any pertaining to the availability and representativeness of available studies, outcomes or other variables.	15-17
Additional analyses	23	Give results of any additional analyses (e.g. sensitivity analyses). If applicable, this should also include any analyses that incorporate aggregate data for studies that do not have IPD. If applicable, summarise the main meta-analysis results following the inclusion or exclusion of studies for which IPD were not available.	15-17, Table 2, Figure 4, Table S4, Figure S3
**Discussion**			
Summary of evidence	24	Summarise the main findings, including the strength of evidence for each main outcome.	17
Strengths and limitations	25	Discuss any important strengths and limitations of the evidence including the benefits of access to IPD and any limitations arising from IPD that were not available.	21-22
Conclusions	26	Provide a general interpretation of the findings in the context of other evidence.	18
Implications	A4	Consider relevance to key groups (such as policy makers, service providers and service users). Consider implications for future research.	18-19

**Funding**			
Funding	27	Describe sources of funding and other support (such as supply of IPD), and the role in the systematic review of those providing such support.	23

Table S2. Assessment of CCA-IMT and carotid plaque.

Study acronym or first author	Location of CCA-IMT measurement	Carotid plaque
**General population**		
AIR	10 mm segment from beginning of bulbar widening	Distinct area with a cIMT >50% thicker than that of neighbouring sites
ARIC	Distal 10 mm defined by BIF origin	If two of three conditions are met: (1) wall shape (protrusion into the lumen, loss of alignment, rough boundary), (2) wall texture (brighter echoes than adjacent boundaries), and (3) wall thickness (cIMT ≥1.5 mm)
CHS	Distal 10 mm of CCA, distal end of CCA defined as beginning of dilatation of bulb with loss of parallel configuration of near and far walls of CCA or as 8 mm proximal to tip of flow divider	Definition based on the greatest wall protrusion (i.e. cIMT) and grading based on lesion surface, echogenicity, and texture characteristics as (1) no plaque (i.e. smooth surface and normal density and morphology), (2) high-risk plaque (i.e. irregular/ulcerated surface, echolucent, or heterogeneous texture), and (3) intermediate-risk plaque (i.e. any other combinations of lesion characteristics)
CMCS-BEIJING	NR	cIMT ≥1.3 mm or focal structure encroaching into arterial lumen of ≥0.5 mm or ≥50% of surrounding cIMT
EVA	NR	Localised echo structures encroaching into the vessel lumen with a distance ≥1 mm between media-adventitia interface and lesion surface facing the lumen
KIHD	10-15 mm section of CCA below bulb	Distinct area either with mineralisation (bright echo, often producing a typical echogenic shadow) or with focal protrusion into the lumen
MESA	Length of 10 mm starting 5-10 mm below bulb	Discrete, focal thickening ≥1.5 mm or ≥50% greater than the surrounding cIMT
NOMAS-INVEST	10-20 mm proximal to tip of flow divider	Focal wall thickening or protrusion into the lumen >50% greater than the surrounding thickness
PIVUS	10-20 mm proximal to bulb	Local thickening of the intima-media by >50% vs. surrounding cIMT
PLIC	5, 10, 20, 25, and 30 mm from bulb	Focal plaque >1.3 mm in longitudinal resolution, lateral, or medial angle
ROTTERDAM	10 mm long segment from beginning of dilatation	Focal widening relative to adjacent segments, with protrusion into the lumen with calcified deposits or both calcified and non-calcified material
SAPHIR	8 mm proximal to tip of flow divider	Grading as (1) normal, (2) vessel wall thickening <1 mm, (3) one minimal plaque ≤2 mm, (4) two moderate plaques ≤3 mm, (5) severe plaque >3 mm, and (6) completely obstructed lumen
**High-risk populations**		
BK REGISTRY	10 mm proximal to bulb	Focal structure encroaching into arterial lumen by ≥50% of surrounding cIMT or thickness >1.2 mm
CSN	Distal 10 mm	cIMT >1.5 mm
IMPROVE	Entire length	cIMT ≥1.5 mm
Kato	Sections of ca. 20-30 mm of CCA just below BIF	cIMT >1 mm
Landecho	10 mm proximal to bulb	Echogenic structures encroaching on the vessel's lumen with a distinct area 50% greater than the cIMT of neighbouring sites
NIGUARDA-MONZINO	Last distal 10 mm of CCA and CCA in entire length	cIMT ≥1.5 mm
**Clinical trials**		
EGE STUDY	Distal 10 mm proximal to bulb and bulb	NR
ENHANCE	10 mm proximal to dilatation	cIMT >1.3 mm

Abbreviations: BIF, carotid bifurcation; CCA, common-carotid artery; CCA-IMT, common-carotid artery intima-media thickness cIMT, carotid intima-media thickness; NR, not reported.

Table S3. Additional characteristics of studies contributing to the analysis.

Study acronym or first author	Ethnicity (white)	BMI (kg/m^2)	SBP (mmHg)	Anti-hypertensive medication (current)	LDL cholesterol (mmol/L)	HDL cholesterol (mmol/L)	Lipid-lowering medication (current)	eGFR (mL/min/ 1.73 m^2)	log hsCRP (mg/L)	Diabetes mellitus (yes)	Smoking (current)
**General population**											
AIR	206 (100)	26 (4)	120 (16)	1 (0)	3.97 (0.98)	1.28 (0.38)	0 (0.0)	75 (9)	0.10 (1.23)	0 (0.0)	43 (21)
ARIC	5,796 (75)	27 (5)	119 (17)	1,853 (24)	3.48 (0.99)	1.37 (0.45)	171 (2.2)	70 (12)	0.91 (1.18)	716 (9.3)	1,852 (24)
CHS	800 (87)	26 (4)	131 (20)	301 (33)	3.22 (0.84)	1.52 (0.42)	31 (3.4)	69 (15)	1.16 (1.21)	88 (9.6)	72 (8)
CMCS-BEIJING	0 (0)	25 (3)	128 (18)	196 (26)	3.33 (0.82)	1.40 (0.31)	76 (10.3)	-	-0.21 (1.15)	42 (5.7)	82 (11)
EVA	769 (100)	25 (4)	130 (17)	193 (25)	4.18 (0.92)	1.66 (0.44)	158 (20.5)	-	0.20 (0.80)	45 (5.9)	59 (8)
KIHD	552 (100)	26 (3)	131 (15)	50 (9)	3.78 (0.94)	1.31 (0.29)	0 (0.0)	86 (14)	0.15 (0.99)	12 (2.2)	136 (25)
MESA	752 (36)	28 (5)	122 (19)	622 (30)	3.01 (0.77)	1.33 (0.39)	252 (12.0)	83 (15)	0.53 (1.16)	124 (6.4)	256 (12)
NOMAS-INVEST	35 (13)	28 (4)	139 (18)	50 (35)	3.31 (0.80)	1.22 (0.37)	16 (15.5)	79 (14)	0.63 (1.28)	31 (21.4)	11 (9)
PIVUS	240 (100)	27 (4)	146 (23)	59 (25)	3.36 (0.85)	1.55 (0.43)	22 (9.2)	77 (14)	0.24 (0.91)	10 (4.2)	14 (6)
PLIC	1,315 (100)	26 (4)	131 (17)	294 (22)	3.68 (0.95)	1.44 (0.39)	118 (9.0)	-	0.30 (1.39)	35 (2.7)	263 (20)
ROTTERDAM	1,193 (99)	26 (3)	131 (20)	235 (19)	-	1.41 (0.35)	8 (0.7)	76 (12)	0.31 (0.99)	53 (4.3)	209 (18)
SAPHIR	917 (100)	27 (4)	137 (17)	109 (12)	3.70 (0.92)	1.58 (0.41)	28 (3.1)	95 (12)	-1.85 (1.00)	19 (2.1)	175 (19)
**High-risk populations**											
BK REGISTRY	0 (0)	25 (3)	121 (15)	191 (90)	2.89 (0.75)	1.09 (0.27)	111 (52.4)	80 (17)	0.18 (1.24)	48 (22.5)	58 (27)
CSN	1,713 (100)	28 (4)	137 (14)	1,360 (81)	3.24 (0.79)	1.33 (0.33)	-	82 (16)	-0.40 (1.39)	116 (11.3)	221 (19)
IMPROVE	1,091 (99)	27 (4)	137 (17)	567 (61)	3.64 (1.05)	1.31 (0.38)	548 (58.9)	-	-	232 (21.0)	125 (11)
Kato	0 (0)	21 (3)	-	34 (35)	2.40 (0.73)	1.27 (0.40)	4 (4.1)	4 (2)	-0.15 (1.56)	-	16 (16)
Landecho	198 (100)	28 (4)	126 (19)	48 (24)	3.84 (0.85)	1.27 (0.32)	25 (12.6)	86 (15)	1.19 (0.93)	14 (7.1)	51 (26)
NIGUARDA-MONZINO	498 (100)	24 (3)	124 (14)	-	4.38 (1.32)	1.39 (0.44)	-	-	-	14 (2.8)	121 (24)
**Clinical trials**											
EGE STUDY	117 (100)	25 (4)	122 (15)	15 (14)	2.88 (0.84)	1.02 (0.32)	10 (9.3)	5 (1)	-0.72 (1.33)	19 (16.2)	21 (18)
ENHANCE	-	27 (5)	123 (13)	-	8.06 (1.76)	1.21 (0.30)	0 (0.0)	79 (13)	0.56 (1.14)	8 (1.3)	174 (29)
**Total**	**16,192 (78)**	**27 (5)**	**126 (19)**	**6,178 (31)**	**3.62 (1.30)**	**1.38 (0.41)**	**1,578 (8.4)**	**75 (17)**	**0.10 (1.33)**	**1,626 (8.0)**	**3,959 (19)**

Continuous variables are expressed as mean (standard deviation) and categorical variables as number (percentage). -, not provided. Abbreviations: BMI, body mass index; eGFR, estimated glomerular filtration rate; HDL, high-density lipoprotein; hsCRP, high-sensitivity C-reactive protein; LDL, low-density lipoprotein; SBP, systolic blood pressure.

Table S4. Sex-specific association between baseline CCA-IMT and incident carotid plaque progressively adjusted for traditional and emerging cardiovascular risk factors.

Level of adjustment	Women			Men		
	OR (95% CI) for incident carotid plaque per SD higher baseline CCA-IMT	P value (χ^2)	I^2 (%)	OR (95% CI) for incident carotid plaque per SD higher baseline CCA-IMT	P value (χ^2)	I^2 (%)
**Primary analysis**	*18 studies; 11,756 participants; 4,228 incident plaques*			*18 studies; 8,980 participants; 3,611 incident plaques*		
Adjusted for age, sex, and trial arm	1.38 (1.24, 1.53)	<0.001 (36.6)	69.0	1.39 (1.31, 1.46)	<0.001 (132.7)	10.8
**Progressive adjustment***	*13 studies; 9,096 participants; 3,304 incident plaques*			*13 studies; 6,496 participants; 2,668 incident plaques*		
Adjusted for age, sex, and trial arm	1.38 (1.21, 1.56)	<0.001 (25.1)	67.1	1.35 (1.28, 1.42)	<0.001 (119.3)	0.0
above + ethnicity	1.38 (1.21, 1.57)	<0.001 (23.9)	68.6	1.36 (1.29, 1.44)	<0.001 (126.6)	0.0
above + smoking status	1.38 (1.21, 1.57)	<0.001 (23.1)	69.4	1.36 (1.28, 1.43)	<0.001 (121.5)	0.0
above + history of diabetes	1.38 (1.20, 1.57)	<0.001 (22.2)	69.8	1.35 (1.28, 1.42)	<0.001 (116.7)	0.0
above + body mass index	1.38 (1.20, 1.58)	<0.001 (21.2)	70.6	1.37 (1.29, 1.44)	<0.001 (120.3)	0.0
above + systolic blood pressure	1.34 (1.19, 1.52)	<0.001 (21.6)	63.5	1.35 (1.27, 1.42)	<0.001 (108.1)	0.0
above + LDL cholesterol	1.32 (1.16, 1.50)	<0.001 (18.7)	64.2	1.33 (1.26, 1.41)	<0.001 (98.8)	0.0
above + HDL cholesterol	1.31 (1.16, 1.48)	<0.001 (18.0)	62.6	1.33 (1.26, 1.41)	<0.001 (97.5)	0.0
above + lipid-lowering medication	1.31 (1.15, 1.48)	<0.001 (17.5)	62.5	1.33 (1.25, 1.40)	<0.001 (96.3)	0.0
above + antihypertensive medication	1.30 (1.14, 1.47)	<0.001 (16.1)	63.3	1.33 (1.25, 1.40)	<0.001 (95.6)	0.0
**Further adjustment for eGFR***	*9 studies; 6,788 participants; 2,701 incident plaques*			*9 studies; 5,071 participants; 2,204 incident plaques*		
Multivariable adjusted†	1.22 (1.02, 1.46)	0.033 (4.6)	65.5	1.31 (1.23, 1.40)	<0.001 (70.6)	0.0
above + eGFR	1.21 (1.01, 1.46)	0.041 (4.2)	65.8	1.31 (1.23, 1.40)	<0.001 (69.8)	0.0
**Further adjustment for hsCRP***	*10 studies; 3,426 participants; 1,156 incident plaques*			*11 studies; 2,836 participants; 1,069 incident plaques*		
Multivariable adjusted†	1.31 (1.14, 1.51)	<0.001 (15.0)	33.2	1.41 (1.28, 1.55)	<0.001 (50.5)	0.0
above + log hsCRP	1.32 (1.16, 1.51)	<0.001 (16.9)	28.8	1.40 (1.27, 1.54)	<0.001 (47.6)	0.0

Analyses for women and men are restricted to the same studies. *Restricted to individuals having information on all variables included in the model. †Adjusted for age at baseline, sex, trial arm, ethnicity, smoking status at baseline, history of diabetes mellitus at baseline, systolic blood pressure at baseline, body mass index at baseline, low-density lipoprotein cholesterol at baseline, high-density lipoprotein cholesterol at baseline, intake of lipid-lowering medication at baseline, and intake of antihypertensive treatment at baseline. Abbreviations: CCA-IMT, common-carotid artery intima-media thickness; CI, confidence interval; eGFR, estimated glomerular filtration rate; HDL, high-density lipoprotein; hsCRP, high-sensitivity C-reactive protein; LDL, low-density lipoprotein; OR, odds ratio; SD, standard deviation.

Figure S1. Location of CCA-IMT measurement.

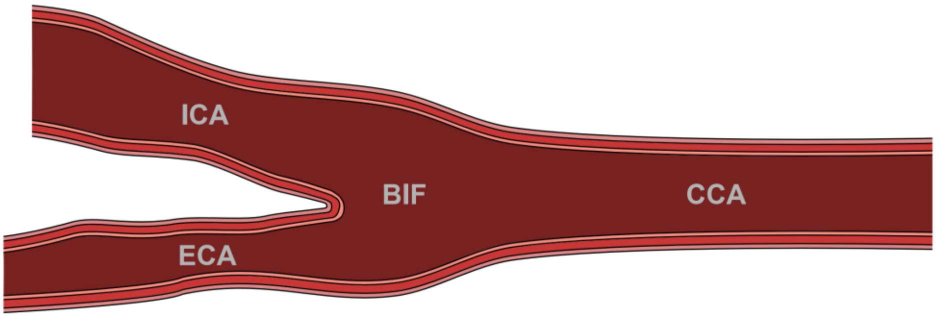

Study acronym or first author	cm from bulbar widening (0, 0.5, 1, 1.5, 2, 2.5, 3)	wall: near	wall: far	side: left	side: right
**General population**					
AIR		X	✓	✓	✓
ARIC		✓	✓	✓	✓
CHS		✓	✓	✓	✓
CMCS-BEIJING		✓	✓	✓	✓
EVA		X	✓	✓	✓
KIHD		X	✓	✓	✓
MESA		✓	✓	✓	✓
NOMAS-INVEST		✓	✓	✓	✓
PIVUS		✓	✓	✓	✓
PLIC		X	✓	✓	✓
ROTTERDAM		✓	✓	✓	✓
SAPHIR		✓	✓	✓	✓
**High-risk populations**					
BK REGISTRY		X	✓	✓	✓
CSN		✓	✓	✓	✓
IMPROVE		X	✓	✓	✓
Kato		X	✓	✓	✓
Landecho		✓	✓	✓	✓
NIGUARDA-MONZINO		✓	✓	✓	✓
**Clinical trials**					
EGE STUDY		X	✓	✓	✓
ENHANCE		X	✓	✓	✓

Abbreviations: BIF, carotid bifurcation; CCA, common-carotid artery; CCA-IMT, common-carotid artery intima-media thickness; ECA, external carotid artery; ICA, internal carotid artery.

Figure S2. Study-specific and overall association between baseline CCA-IMT and incidence of carotid plaque.

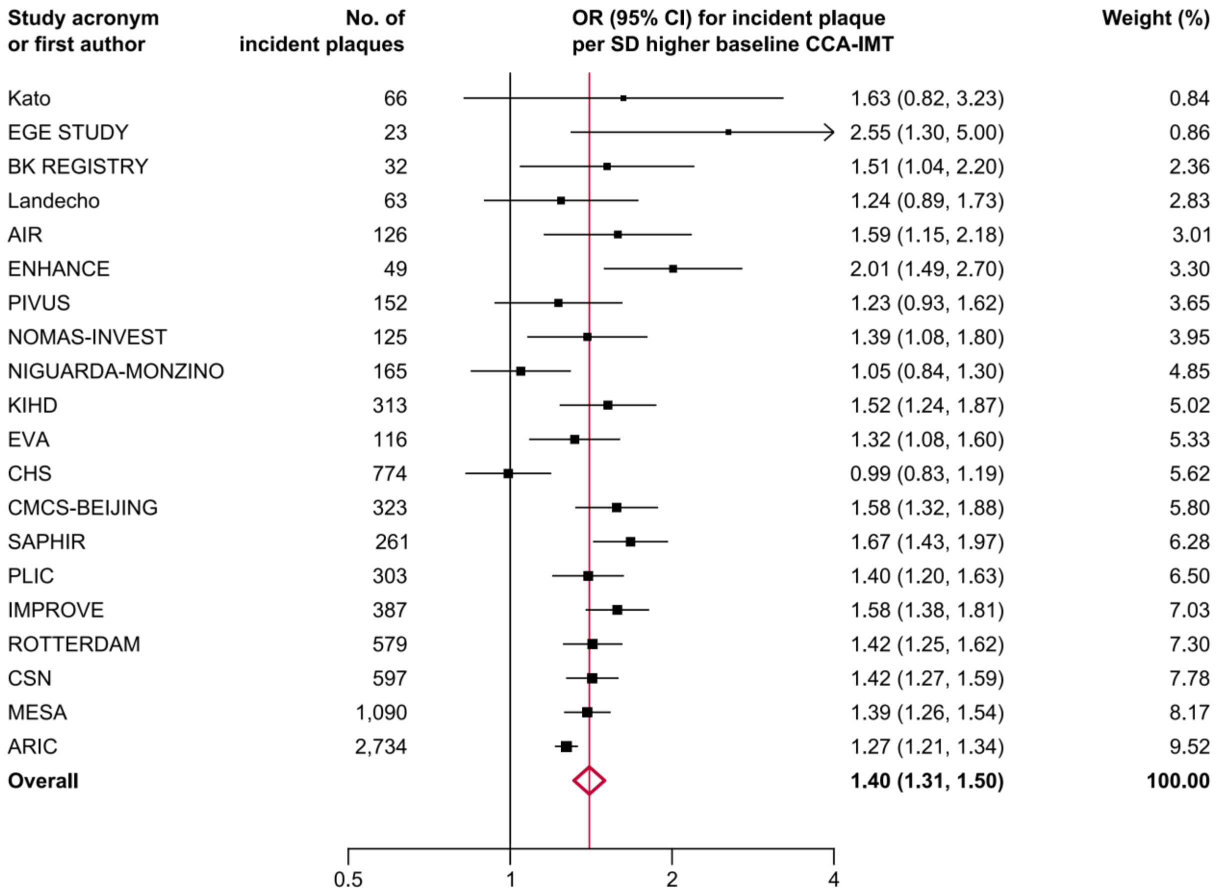

The model has been adjusted for age at baseline, sex, and trial arm. Abbreviations: CCA-IMT, common-carotid artery intima-media thickness; CI, confidence interval; OR, odds ratio; SD, standard deviation.

Figure S3. Subgroup analysis of association between baseline CCA-IMT and incidence of carotid plaque by median duration of follow-up.

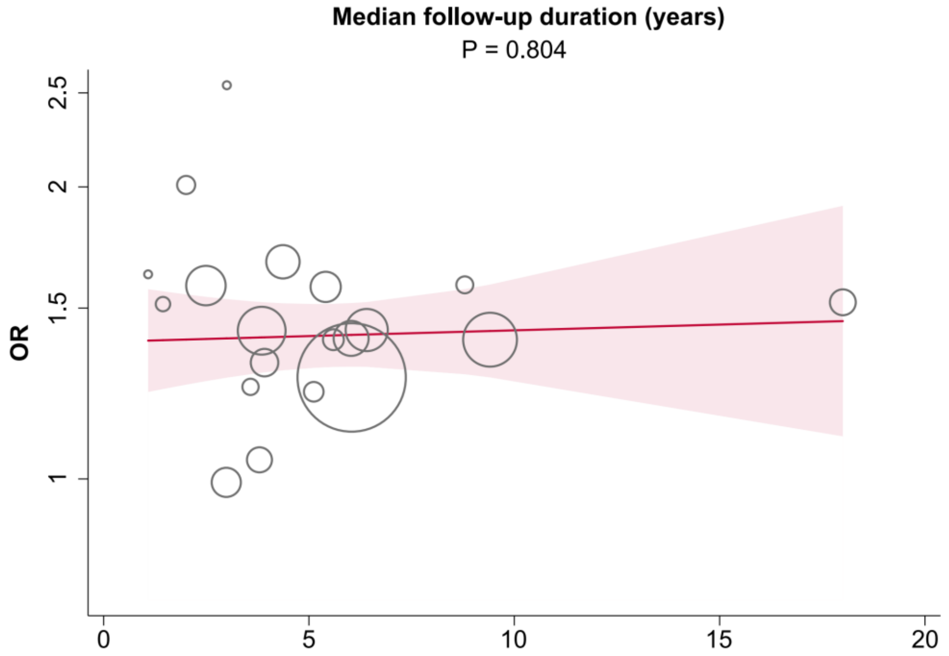

The P value is derived from meta-regression. Each bubble depicts a study. The centres of the bubbles indicate point estimates for odds ratios for development of carotid plaque per standard deviation higher level of baseline common-carotid artery carotid intima-media thickness plotted against median durations of follow-up for each study. Odds ratios are adjusted for age at baseline, sex, and trial arm. The sizes of the bubbles are proportional to the inverse variances of the estimates. Abbreviations: OR, odds ratio.

Figure S4. Flow diagram literature search.

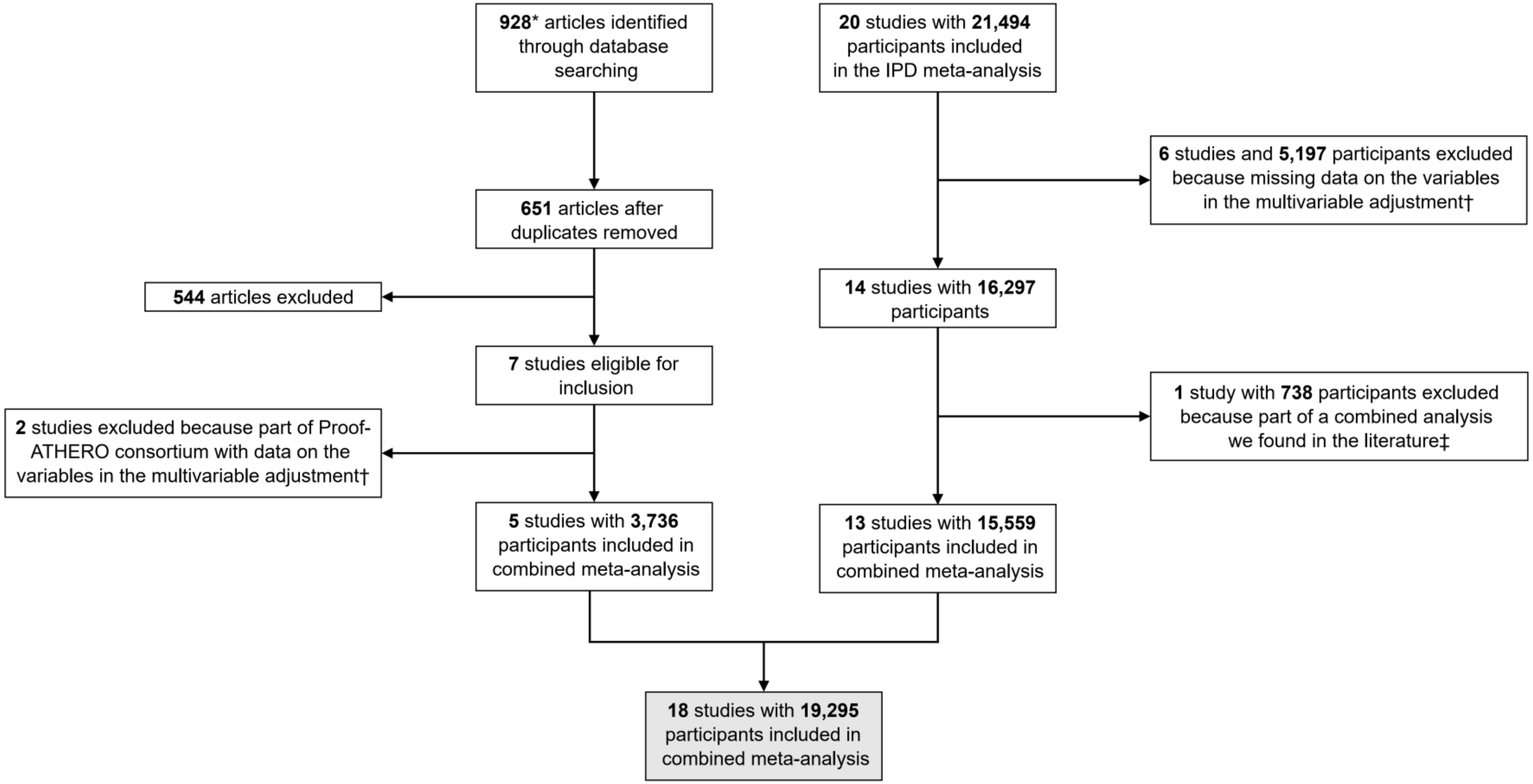

*472 from PubMed and 456 from Web of Science. †The multivariable adjustment included the variables age at baseline, sex, trial arm, ethnicity, smoking status at baseline, history of diabetes mellitus at baseline, systolic blood pressure at baseline, body mass index at baseline, low-density lipoprotein cholesterol at baseline, high-density lipoprotein cholesterol at baseline, intake of lipid-lowering medication at baseline, and intake of antihypertensive medication at baseline. ‡The CMCS-BEIJING study was excluded from the individual-participant-data meta-analysis since we used aggregated data of both the CMCS and People's Republic of China-United States Collaborative Study in Cardiovascular and Cardiopulmonary Epidemiology study. Abbreviations: IPD, individual-participant-data; Proof-ATHERO, Prospective Studies of Atherosclerosis.
